# 2*H*‐Dinaphthopentacene: A Polycyclic Aromatic Hydrocarbon Core for Metal‐Free Organic Sensitizers in Efficient Dye‐Sensitized Solar Cells

**DOI:** 10.1002/advs.201700099

**Published:** 2017-04-25

**Authors:** Yameng Ren, Jiao Liu, Aibin Zheng, Xiandui Dong, Peng Wang

**Affiliations:** ^1^ Changchun Institute of Applied Chemistry Chinese Academy of Sciences Changchun 130022 China; ^2^ University of Chinese Academy of Sciences Beijing 100049 China; ^3^ Department of Chemistry Zhejiang University Hangzhou 310028 China

**Keywords:** charge transfer, dyes, excited states, polycyclic aromatic hydrocarbon, solar cells

## Abstract

Continuous studies on the use of a polycyclic aromatic hydrocarbon as the central block of an organic photosensitizer have brought forth a new opportunity toward efficiency enhancement of dye‐sensitized solar cells (DSCs). In this paper, a nonacyclic aromatic hydrocarbon 9,19‐dihydrodinaphtho[3,2,1‐*de*:3′,2′,1′‐*op*]pentacene, tethered with four 4‐hexylphenyl solubilizing groups is reported. The novel chromophore 9,9‐19‐19‐tetrakis(4‐hexylphenyl)‐9,19‐dihydrodinaphtho[3,2,1‐*de*:3′,2′,1′‐*op*]pentacene is further functionalized with diarylamines and 4‐(7‐ethynylbenzo[*c*][1,2,5]thiadiazol‐4‐yl)benzoic acid to produce two donor–acceptor (D–A) organic photosensitizers, achieving good power conversion efficiencies up to 10.2% in DSCs.

## 1 Introduction

Organic small molecules with polycyclic aromatic hydrocarbons (PAH) as the kernel segments are in general characteristic of high molar absorption coefficient, large luminescence yield, and excellent carrier mobility, triggering their extensive utilizations as light‐emitters, pigments, and optoelectronic materials.^1^ In recent studies, we and other groups have selected the highly emissive *N*‐annulated perylene (NP)^2^ to construct organic D–A dyes with desirable anti‐aggregation capacity,^3^, ^4^ achieving high power conversion efficiencies (PCEs) up of 13.0% without use of any coadsorbate.[[qv: 3h]] However, it should be perceived that NP is synthesized from 1‐nitroperylene, which can only be obtained from the nitration reaction of perylene at an 30% yield due to the remarkable formation of 3‐nitroperylene.[[qv: 2a,b]]

**Figure 1:**
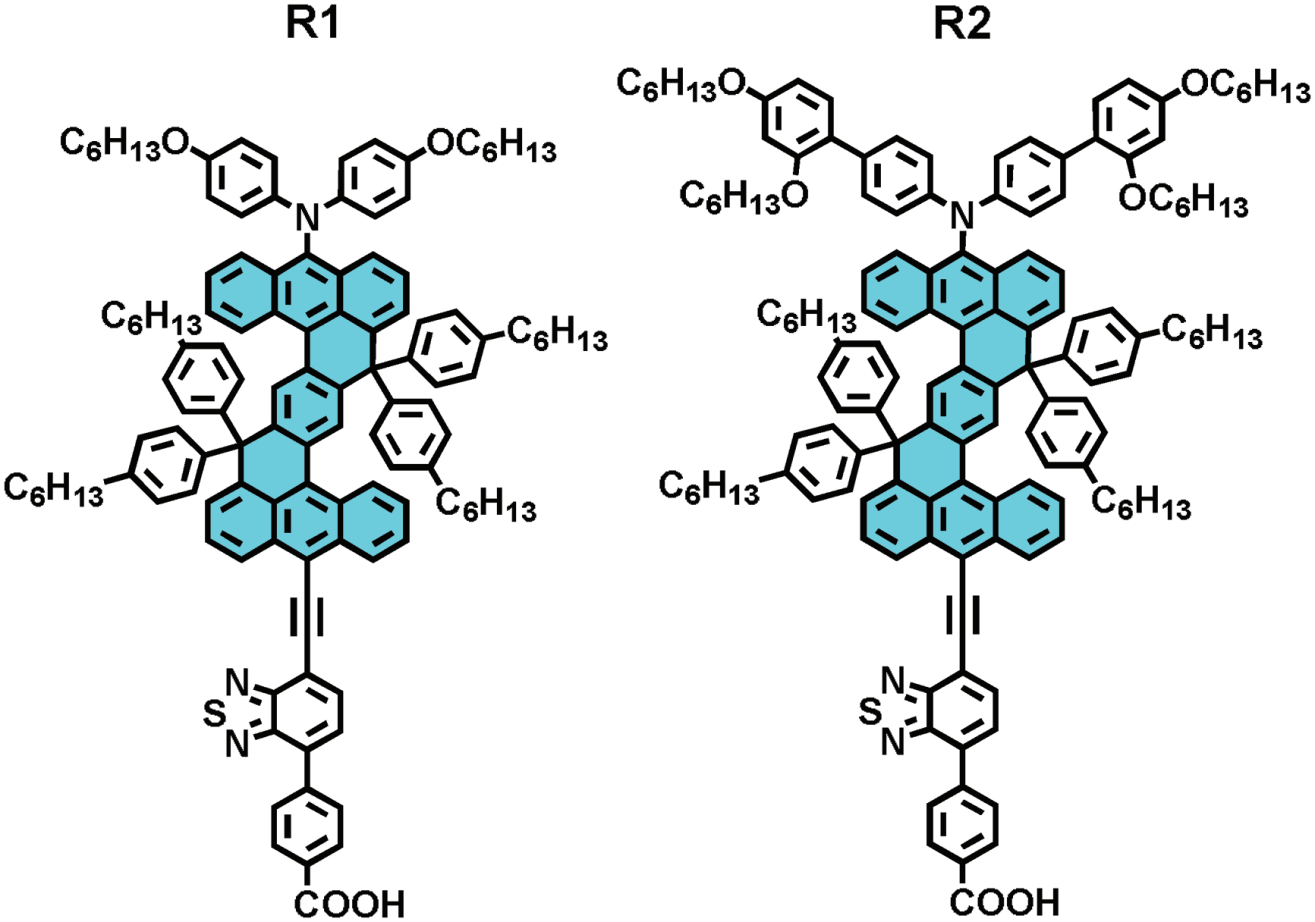
Molecular structures of **R1** and **R2** characteristic of a polycyclic aromatic hydrocarbon DNP, which is filled with the color of cyan.

Thereby, we have turned our interest to anthracene, which has been previously used as the building block of organic dyes for dye‐sensitized solar cells (DSCs), but all these dyes only displayed a low or moderate PCE.^5^ In our initial exploration of anthracene related chemistries, we envision a nonacyclic aromatic hydrocarbon, 9,19‐dihydrodinaphtho[3,2,1‐*de*:3′,2′,1′‐*op*]pentacene (DNP, **Figure** **1**), which can be decorated with four 4‐hexylphenyl groups to allow for a soluble target compound. Note that other noncyclic conjugated units have been previously exploited for organic solar cells.^6^ As a preliminary test of the potential of DNP for optoelectronic materials, we will resort to the same design strategy for the NP and zinc‐porphyrin based dyes (C289, SC‐3, GY50, and SM315 in Figure S1, Supporting Information),[[qv: 3k,l,7,8]] to functionalize DNP with diarylamine and 4‐(7‐ethynylbenzo[*c*][1,2,5]thiadiazol‐4‐yl)benzoic acid for organic photosensitizers **R1** and **R2** (Figure 1).

## 2 Results and Discussion

**Scheme 1:**
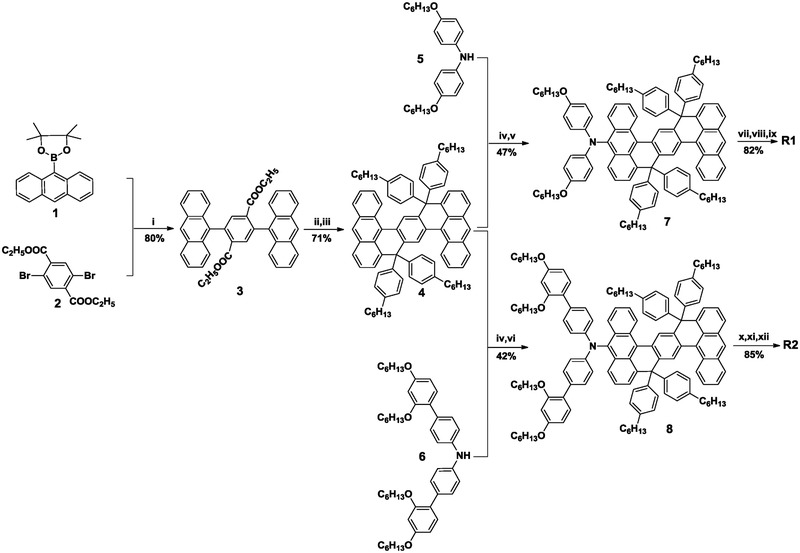
Synthetic routes to **R1** and **R2**. Reagents and conditions: (i) Pd(OAc)_2_, Sphos, K_3_PO_4_, dioxane/H_2_O (*v*/*v*, 5/1), reflux, 12 h; (ii) (4‐hexylphenyl)magnesium bromide, THF, reflux, 6 h; (iii) Amberlyst 15, toluene, reflux, 6 h; (iv) NBS, THF, R.T., 10 min; (v) bis(4‐(hexyloxy)phenyl)amine, Pd_2_(dba)_3_, P(*t*‐Bu)_3_, NaO*t*Bu, toluene, reflux, 12 h; (vi) bis(2′,4′‐bis(hexyloxy)‐[1,1′‐biphenyl]‐4‐yl)amine, Pd_2_(dba)_3_, P(*t*‐Bu)_3_, NaO*t*Bu, toluene, reflux, 12 h; (vii) NBS, THF, R.T., 1 h; (viii) butyl 4‐(7‐ethynylbenzo[*c*][1,2,5]thiadiazol‐4‐yl)benzoate, Pd_2_(dba)_3_, P(*t*‐Bu)_3_, Cs_2_CO_3_, dioxane, reflux, 12 h; (ix) KOH, THF/H_2_O (*v*/*v*, 3/1), reflux, 9 h; then phosphoric acid; (x) NBS, THF, R.T., 1 h; (xi) butyl 4‐(7‐ethynylbenzo[*c*][1,2,5]thiadiazol‐4‐yl)benzoate, Pd_2_(dba)_3_, P(*t*‐Bu)_3_, Cs_2_CO_3_, dioxane, reflux, 12 h; (xii) KOH, THF/H_2_O (*v*/*v*, 3/1), reflux, 9 h; then phosphoric acid.

The synthetic routes to **R1** and **R2** are outlined in **Scheme** **1**. First, the key intermediate diethyl 2,5‐di(anthracen‐9‐yl)terephthalate (**3**) was prepared in good yield via the Suzuki‐Miyaura cross‐coupling of 2‐(anthracen‐9‐yl)‐4,4,5,5‐tetramethyl‐1,3,2‐dioxaborolane (1) with diethyl 2,5‐dibromoterephthalate (2), by use of the highly active phosphine ligand 2‐(2′,6′‐dimethoxybiphenyl)dicyclohexylphosphine (Sphos).^9^ Thereafter, we implemented a carbonyl addition reaction of **3** using (4‐hexylphenyl)magnesium bromide to acquire a bis‐tertiary alcohol intermediate, which underwent intramolecular Friedel‐Crafts cyclization with the aid of solid acid catalyst Amberlyst 15 to afford 9,9‐19‐19‐tetrakis(4‐hexylphenyl)‐9,19‐dihydrodinaphtho[3,2,1‐*de*:3′,2′,1′‐*op*]pentacene (**4**, THPDNP). Our DNP synthesis adopted the synthetic strategy for fluorene first proposed by Holmes and co‐workers^10^ Then, we performed bromination of **4** at room temperature to get a bromide, which was further cross‐coupled with bis(4‐(hexyloxy)phenyl)amine (DPA, **5**)[[qv: 3a]] or bis(2′,4′‐bis(hexyloxy)‐[1,1′‐biphenyl]‐4‐yl)amine (DBPA, **6**)^11^ via the Buchwald‐Hartwig cross‐coupling reaction to afford *N*,*N*‐bis(4‐(hexyloxy)phenyl)‐9,9,19,19‐tetrakis(4‐hexylphenyl)‐9,19‐dihydrodinaphtho[3,2,1‐*de*:3′,2′,1′‐*op*]pentacen‐5‐amine (DPA‐THPDNP, **7**) or *N*,*N*‐bis(2′,4′‐bis(hexyloxy)‐[1,1′‐biphenyl]‐4‐yl)‐9,9,19,19‐tetrakis(4‐hexylphenyl)‐9,19‐dihydrodinaphtho[3,2,1‐*de*:3′,2′,1′‐*op*]pentacen‐5‐amine (DBPA‐THPDNP, **8**). Subsequently, we performed mono‐bromination of **7** or **8** at room temperature to obtain a bromide, which was further cross‐coupled with butyl 4‐(7‐ethynylbenzo[*c*][1,2,5]thiadiazol‐4‐yl)benzoate[[qv: 3d]] using the Sonogashira reaction to produce an esterified dye precursor. Eventually, the esters were hydrolyzed with KOH as the catalyst, and the hydrolyzate were thoroughly acidified with diluted phosphoric acid aqueous solution to afford the desired dyes **R1** and **R2**.

**Figure 2:**
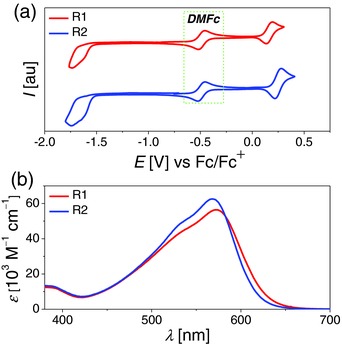
a) Cyclic voltammograms of **R1** and **R2** in THF. Scan rate: 5 mV s^−1^. Decamethylferrocene (DMFc) was added as the internal reference and all potentials were further calibrated with the standard redox couple ferrocene/ferrocenium (Fc/Fc^+^). b) Stationary UV–vis spectroscopies of **R1** and **R2** in THF (10 µm).

To understand the energy level evolution of the highest occupied molecular orbital (HOMO) and lowest unoccupied molecular orbital (LUMO) during the derivatization of THPDNP, by sequentially tethering auxiliary electron‐donor DPA (or DBPA) and electron‐acceptor 4‐(7‐ethynylbenzo[*c*][1,2,5]thiadiazol‐4‐yl)benzoic acid (EBTBA), we first performed electrochemical measurements of these anthracene‐based molecules under an inert atmosphere of nitrogen. Cyclic voltammograms (CVs) are presented in **Figure** **2**a and Figure S2a (Supporting Information), and the derived energy levels are tabulated in **Table** **1**. It is found that the attachment of DPA (or DBPA) to THPDNP brings about a LUMO downwards movement slightly, but has a profound influence on the HOMO upward movement, leading to the shrinkage of energy gap. Furthermore, it is figured out that with respect to **R1** using DPA‐THPDNP as electron‐donor, the DBPA‐THPDNP based dye **R2** is born with a 0.08 eV deeper HOMO energy level. In consistence with a previous work,^12^ our theoretical calculations have also revealed that DPA is a stronger donor group than DBPA. In addition, these two dyes own the same LUMO energy level of −3.34 eV versus vacuum. On the whole, the transformation of electron‐donor from DPA‐THPDNP to DBPA‐THPDNP gives birth to a little bit larger LUMO/HOMO energy gap ($\Delta E_{\mathrm{L/H}}^{\mathrm{CV}}$). It is thus reasonable for us to detect a 4 nm blueshift of maximum absorption wavelength ($\lambda_{\mathrm{ABS},\;\mathrm{MAX}}^{\mathrm{MEAS}}$) for **R2** from Figure 1b. However, the end‐capping of THPDNP with DBPA produces an augmented maximum molar absorption coefficients ($\varepsilon_{\mathrm{MAX}}^{\mathrm{MEAS}}$) of 62.6 × 10^3^ M^−1^ cm^−1^ for **R2**, in contrast to that of 56.4 × 10^3^ M^−1^ cm^−1^ for **R1** with DPA.

**Table 1:** Energy levels and photophysical data

Dyea)	$E_{L}^{\mathrm{CV}}$ [eV]	$E_{L}^{\mathrm{B3LYP}}$ [eV]	$E_{H}^{\mathrm{CV}}$ [eV]	$E_{H}^{\mathrm{B3LYP}}$ [eV]	$\lambda_{\mathrm{ABS},\;\mathrm{MAX}}^{\mathrm{MEAS},\mathrm{THF}}$ [nm]	$\lambda_{\mathrm{ABS},\;\mathrm{MAX}}^{\mathrm{TD‐MPW1K},\mathrm{THF}}$ [nm]	$\tau_{\mathrm{EX}}^{\mathrm{THF}}$ [ps]	$\varepsilon_{\mathrm{ABS},\;\mathrm{MAX}}^{\mathrm{MEAS},\mathrm{THF}}$ [10^3^ M^−1^ cm^−1^]
THPDNP	−2.80	−2.48	−5.21	−5.19	485	465	/	16.4
DPA‐THPDNP	−2.84	−2.52	−4.94	−4.93	520	504	/	20.9
DBPA‐THPDNP	−2.86	−2.54	−5.04	−5.02	514	494	/	30.3
**R1**	−3.34	−3.09	−4.97	−4.95	572	580	468	56.4
**R2**	−3.34	−3.09	−5.05	−5.03	568	577	1296	62.6

^a)^ Electrochemically measured frontier orbital energies ($E_{L}^{\mathrm{CV}}$and$E_{H}^{\mathrm{CV}}$) versus vacuum are calculated via $E\;=\;-\;4.\mathrm{88}\;-\;{\mathrm{eE}}_{\mathrm{onset}}$, herein $E_{\mathrm{onset}}$ is the onset oxidation and reduction potentials (Figure 2a and Figure S2a, Supporting Information) of a molecule at the ground‐state in THF. H and L stand for HOMO and LUMO, respectively. Energy levels of LUMO and HOMO ($E_{L}^{\mathrm{B3LYP}}$and $E_{H}^{\mathrm{B3LYP}}$) are computed at the B3LYP/6‐311G(d,p) level of theory for a dye molecule in THF. Maximum absorption wavelength ($\lambda_{\mathrm{ABS},\;\mathrm{MAX}}^{\mathrm{MEAS}}$) and maximum molar absorption coefficient ($\varepsilon_{\mathrm{ABS},\;\mathrm{MAX}}^{\mathrm{MEAS},\mathrm{THF}}$) are derived from electronic absorption spectroscopies of THF solutions. Maximum absorption wavelength ($\lambda_{\mathrm{ABS},\;\mathrm{MAX}}^{\mathrm{TD‐MPW1K}}$) is calculated at the TD‐MPW1K/6‐311G(d,p) level of theory for a dye molecule in THF. The equilibrium excited state lifetimes ($\tau_{\mathrm{EX}}^{\mathrm{THF}}$) are derived from Figure S6 (Supporting Information).

As noted from Table 1 and Figure S4 (Supporting Information), our theoretical calculations can finely reproduce the relative tendencies of electronic energy level and maximum absorption wavelength. Time‐dependent density functional theory (TDDFT) calculations at the TD‐MPW1K/6‐311G(d,p) level have unlocked that the S1←S0 vertical electronic transitions to LUMO of these two dyes stem mainly from HOMO, manifesting an explicit intramolecular charge‐transfer character (Figure S5, Supporting Information). As presented in Figure S6 (Supporting Information) the lifetimes of **R1** and **R2** at the equilibrium excited state in tetrahydrofuran (THF) are ≈0.5 and ≈1.3 ns, respectively. It is valuable to note that the selection of ancillary electron‐donor can have a remarkable influence on the excited state lifetime. This point should be judiciously taken into account in the future design of organic photosensitizers.

**Figure 3:**
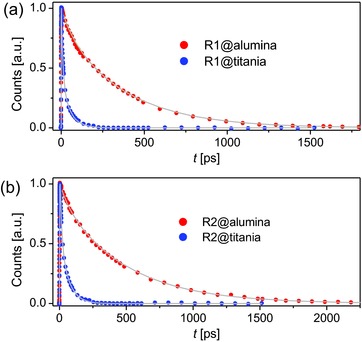
a,b) Up‐converted PL traces probed at 850 nm of dye molecules grafted on mesoporous alumina and titania films. The grey fitting lines are also included. Pump wavelength: 490 nm; pulse fluence: 28 µJ cm^−2^.

The yields of electron injection (φ_ei_) from excited‐state dye molecules to titania were evaluated with the femtosecond fluorescence up‐conversion technique.^13^ First, a mesoporous alumina film grafted with dye molecules and further infiltrated with a Co‐bpy electrolyte (for it recipe, see the Experimental Section) was exposed to femtosecond pulse excitation, and the amplitude‐averaged lifetimes $\bar{\tau }(A)$ of photoluminescence decays (red dots in **Figure** **3**) due to the radiative and nonradiative deactivation of excited‐state dye molecules are 316 ps for **R1** and 423 ps for **R2**. Furthermore, the replacement of alumina with titania induced a distinct shortening of amplitude‐averaged lifetimes $\bar{\tau }(T)$ (23 ps for **R1** and 31 ps for **R2**), indicative of electron injection at the energy‐offset dye‐titania interface (blue dots in Figure 3). The φ_ei_ values, being 92% for **R1** and 93% for **R2**, were calculated via Equation (1)

(1) $$\phi_{\mathrm{ei}}=1-\bar{\tau }(T)/\bar{\tau }(A)$$

**Figure 4:**
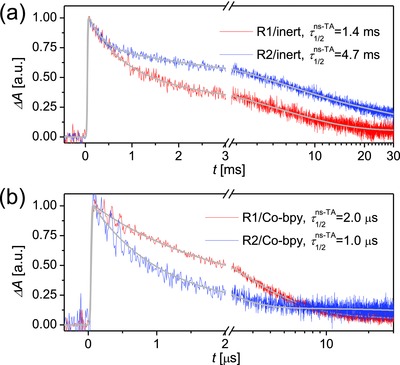
a,b) Absorption transients of dye‐grafted titania films in combination with an inert electrolyte made from 0.5 m TBP and 0.1 m LiTFSI in acetonitrile and a Co‐bpy electrolyte. Excitation wavelength: 629 nm for **R1**/inert; 621 nm for **R2**/inert; 630 nm for **R1**/Co‐bpy; 626 nm for **R2**/Co‐bpy. Probe wavelength: 785 nm. Pulse fluence: 20 µJ cm^−2^. The solid gray lines are multiexponential fittings.

Next, a nanosecond laser flash photolysis spectrometer was employed to measure the kinetics of dual‐path charge‐transfer reactions of photooxidized dye molecules (D^+^) either with mobile electrons in titania or with cobalt(II) ions in the electrolyte. Compared to their neutral forms, the oxidized states of both **R1** and **R2** possess strong electronic absorption in the near‐infrared region (Figure S7, Supporting Information). Thereby, a probe light at 785 nm was used in the kinetic measurements and the excitation wavelengths were also elaborately selected in terms of an optical density of ≈0.5 of dye‐grated titania films, ensuring a similar electron distribution profile in the testing samples. The absorption traces could be nicely fitted with multi‐exponential functions for convenient determination of half‐reaction times ($\tau_{\mathrm{1/2}}^{\mathrm{ns‐TA}}$). When an inert electrolyte composed of 0.1 m lithium bis(trifluoromentylsulfonyl)imide (LiTFSI) and 0.5 m 4‐tert‐butylpyridine (TBP) dissolved in acetonitrile was applied, we recorded decay signals (**Figure** **4**a) on a millisecond time domain, which are related to the electron transfer reaction between oxidized dye molecules and charged titania. It is noted that using a bulky electron‐donor in **R2** induces a desirable larger half‐reaction time ($\tau_{\mathrm{1/2}}^{\mathrm{ns‐TA}}$), which may be accounted for by a larger distance from the positive charge on D^+^ to titania, and/or a smaller tile angle of **R2** on the surface of titania. As presented in Figure 4b, the use of a Co‐bpy electrolyte brought forth much faster absorption decays in the microsecond time domain, suggesting the occurrence of hole injection from D^+^ to cobalt(II) ions. An elongated $\tau_{\mathrm{1/2}}^{\mathrm{ns‐TA}}$ for R1 relative to R2 should be ascribed to a reduced driving force brought about by an uplifted HOMO energy level. Overall, the hole injection yields (φ_hi_) are pretty high, being 99% and 100% for **R1** and **R2**, which are calculated via equation

(2) $$\phi_{\mathrm{hi}}\;=\;1\;-\;\tau_{\mathrm{1/2}}^{\mathrm{Co‐bpy}}/\tau_{\mathrm{1/2}}^{\mathrm{inert}}$$

**Figure 5:**
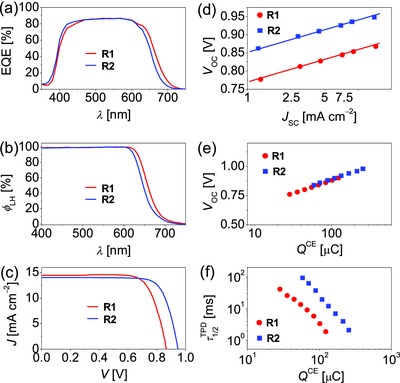
a) External quantum efficiencies (EQEs) at a set of wavelengths (λ) of incident monochromatic lights for dyes R1 and R2 self‐organized on the surface of a bilayer film [(4.5+5.0) µm thick] of mesoporous and microporous titania. b) Wavelength‐dependent light‐harvesting yields (φ_*LH*_) for a single layer (8.0 µm thick) of mesoporous titania grafted with dye molecules. c) Current–voltage (*J*–*V*) curves recorded under the simulated AM1.5G sunlight (100 mW cm^−2^). The aperture area of black metal mask: 0.160 cm^2^. d) Dependence of open‐circuit photovoltage (*V_OC_*) on short‐circuit photocurrent density (*J_SC_*). The solid fitting lines are also included. e) Charge extracted from a dye‐grafted titania film (*Q^CE^*) as a function of open‐circuit photovoltage (*V_OC_*). f) Plots of half‐lifetime ($\tau_{\mathrm{1/2}}^{\mathrm{TPD}}$) of electrons in the conduction band and traps under the conduction band of titania versus *Q^CE^*.

The external quantum efficiencies (EQEs) of DSCs made with a dye‐grafted bilayer titania film and a Co‐bpy electrolyte were measured with monochromatic lights at an interval of 10 nm and a white light bias (10 mW cm^−2^). For the details of cell fabrication, see the Experimental Section. As illustrated in **Figure** **5**a, the photocurrent action spectra of **R1** and **R2** both exhibit a maximum of ≈86%. An ≈17 nm blueshifting of the onset wavelength of photocurrent response was detected for **R2** compared to **R1**, which is in rough accord with the wavelength dependent light‐harvesting yields (φ_LH_) values included in Figure 5b. The photocurrent density–voltage (*J*–*V*) characteristics recorded at an irradiance of 100 mW cm^−2^ simulated AM1.5 sunlight are presented in Figure 5c. The averaged cell parameters of four cells made with each dye are collected in **Table** **2**. Dye **R1** exhibits a short‐circuit photocurrent density (*J*_SC_) of 14.47 mA cm^−2^, an open‐circuit photovoltage (*V*_OC_) of 867 mV, and a fill factor (FF) of 74.6%, yielding a PCE of 9.4%. In good agreement with φ_LH_ and the integrals of EQEs over the standard AM1.5G emission spectrum (ASTM G173‐03), dye **R2** outputs a slightly reduced *J*_SC_ of 14.00 mA cm^−2^, but an enlarged *V*_OC_ of 948 mV and an excellent FF of 77.2%, affording an enhanced PCE of 10.2%. We observed negligible PCE degradation for cells left on the benchtop for over three months, while the stability tests under accelerated aging under the light and thermal dual stress should be performed in the future study. We also measured *J*–*V* curves at a set of light irradiances and plotted *V*_OC_ as a function of *J*_SC_ as depicted in Figure 5d. From the fitting curves, we note that at a given *J*_SC_ there is an ≈85 mV higher *V*_OC_ for **R2** compared to **R1**.

**Table 2:** Photovoltaic parameters of four cells measured under the simulated AM1.5G sunlight (100 mW cm^−2^)

Dyea)	$J_{SC}^{EQE}$ [mA cm^−2^]	$J_{SC}^{}$ [mA cm^−2^]	V_*OC*_ [mV]	FF [%]	PCE [%]
**R1**	14.59 ± 0.08	14.47 ± 0.09	867 ± 2	74.6 ± 0.2	9.4 ± 0.2
**R2**	14.03 ± 0.04	14.00 ± 0.03	948 ± 3	77.2 ± 0.1	10.2 ± 0.1

^a)^ $J_{\mathrm{SC}}^{\mathrm{EQE}}$ was computed via wavelength integration of the product of the EQE curve measured at the short‐circuit and the standard AM1.5G emission spectrum (ASTM G173‐03).

To figure out the interfacial energetic and dynamic origins of *V*_OC_ variation,^14^ we further performed the charge extraction (CE)^15^ and transient photovoltage decay (TPD)^16^ measurements. Figure 5e shows a very much similar profile of charges stored in titania (*Q* ^CE^) as a function of *V*_OC_, which suggests that the titania films in these two cells possess the same conduction‐band edge (CBE) and the alike distribution of trap states under CBE. However, it can be noted from Figure 5f that at a given*Q* ^CE^, the cell with **R2** bears a significantly elongated half lifetime ($\tau_{\mathrm{1/2}}^{\mathrm{TPD}}$) for electrons in titania, with respect to the **R1** cell, giving an explicit clue on its enlarged *V*_OC_. Moreover, the loading amounts (*c*_m_) of dye molecules on titania were also measured with visible spectrometry, being 1.92 × 10^−8^ mol cm^−2^ µm^−1^ for **R1** and 1.73 × 10^−8^ mol cm^−2^ µm^−1^ for **R2**. Obviously, the adverse impact^17^ of a relatively lower *c*_m_ on *V*_OC_ is over compensated with the bulky electron‐donor in **R2**.^18^

## 3 Conclusions

In summary, we have synthesized two metal‐free organic dyes characteristic of a PAH, 2*H*‐dinaphthopentacene. Dye **R2** with a bulky auxiliary diarylamine electron‐donor has reached an excellent PCE of 10.2% under the AM1.5G full sunlight, owing to the effectively attenuated interfacial charge recombination and a significantly improved photovoltage. Our work should encourage further molecular engineering of anthracene‐based dyes and stimulate active explorations of polycyclic optoelectronic materials with anthracene as the basic building block in other fields.

## 4 Experimental Section

*Materials*: LiTFSI, EMITFSI, DMFc, Fc, TBP, tris(dibenzylideneacetone)dipalladium (Pd_2_(dba)_3_), [1,1′‐bis(diphenylphosphino)ferrocene]dichloropalladium(II) (Pd(dppf)Cl_2_), palladium(II) acetate (Pd(OAc)_2_), 2‐dicyclohexylphosphino‐2′,6′‐dimethoxybiphenyl (Sphos), tris(1,1‐dimethylethyl)phosphine (P(*t*‐Bu)_3_), *N*‐bromosuccinimide (NBS), (triisopropylsilyl)acetylene, sodium *tert*‐butoxide (NaO*t*‐Bu), potassium hydroxide (KOH), potassium phosphate (K_3_PO_4_), diethyl 2,5‐dibromoterephthalate, 4,4,4′,4′,5,5,5′,5′‐octamethyl‐2,2′‐bi(1,3,2‐dioxaborolane), and cesium carbonate (Cs_2_CO_3_) were purchased from Sigma‐Aldrich and used without further purification. Toluene, THF, dioxane, diisopropylamine, acetonitrile, ethanol, and chloroform were dried and distilled before use. (4‐Hexylphenyl)magnesium bromide^19^ was synthesized according to the literature procedure. Other chemicals were purchased and used without further purification. The synthetic routes to **R1** and **R2** are illustrated in Scheme 1 and preparation details are described as follows.

*Diethyl 2,5‐Di(Anthracen‐9‐yl)Terephthalate (* ***3*** *)*: In a three‐neck round‐bottom flask was dissolved 2‐(anthracen‐9‐yl)‐4,4,5,5‐tetramethyl‐1,3,2‐dioxaborolane (1) (6.0 g, 19.73 mmol) and diethyl 2,5‐dibromoterephthalate (2) (3.0 g, 7.89 mmol) in dioxane/H_2_O (24 mL, v/v, 5/1). Then Pd(OAc)_2_ (89 mg, 0.39 mmol), Sphos (160 mg, 0.39 mmol), and K_3_PO_4_ (8.37 g, 39.45 mmol) were added to the reaction mixture under argon, which was refluxed for 12 h. The mixture was extracted three times with chloroform before the organic phase was washed with water and dried over anhydrous sodium sulfate. After solvent removal under reduced pressure, the crude product was purified by recrytallization (toluene) to yield a yellow solid as the desired product **3** (3.63 g, 80% yield). ^1^H NMR (400 MHz, CDCl_3_, δ): 8.59 (s, 2H), 8.25 (s, 2H), 8.12 (d, *J* = 7.6 Hz, 4H), 7.71 (d, *J* = 8.4 Hz, 4H), 7.55–7.47 (m, 8H), 3.67–3.61 (m, 4H), 0.31 (t, *J* = 7.1 Hz, 6H). ^13^C NMR (100 MHz, CDCl_3_, δ): 166.29, 139.34, 135.82, 135.12, 134.96, 131.41, 130.24, 129.18, 128.69, 128.37, 127.00, 126.34, 126.00, 125.32, 60.97, 13.01. HR‐MS (MALDI‐TOF) *m*/*z* calcd. for (C_40_H_30_O_4_): 574.21441. Found: 574.21285. Anal. Calcd. for C_40_H_30_O_4_: C, 83.60%; H, 5.26%; Found: C, 83.61%; H, 5.25%.

*9,9,19,19‐Tetrakis(4‐Hexylphenyl)‐9,19‐Dihydrodinaphtho[3,2,1‐De:3′,2′,1′‐Op]pentacene (* ***4*** *)*: In a dried Schlenk tube was dissolved **3** (3.5 g, 6.09 mmol) in THF (15 mL), and (4‐hexylphenyl)magnesium bromide (18.27 mL, 2 m in THF, 36.54 mmol) was added in one portion via syringe. The mixture was slowly warmed up and stirred at reflux under argon for 6 h. Water was slowly added to terminate the reaction and the mixture was poured into cold 1 m hydrochloric acid aqueous solution. The mixture was extracted three times with chloroform before the organic phase was washed with water and dried over anhydrous sodium sulfate. After solvent removal under reduced pressure, the residue tertiary alcohol was used in the next reaction directly. In a dried Schlenk tube were dissolved the above tertiary alcohol, Amberlyst 15 (1.50 g) in 25 mL toluene, which was refluxed for 6 h. The mixture was extracted three times with chloroform before the organic phase was washed with water and dried over anhydrous sodium sulfate. After solvent removal under reduced pressure, the crude product was purified by recrytallization (toluene/petroleum ether 60–90 °C) to yield a yellow solid as the desired product **4** (4.74 g, 71% yield). ^1^H NMR (400 MHz, CDCl_3_, δ): 8.31 (s, 2H), 8.00 (d, *J* = 8.9 Hz, 2H), 7.94 (s, 2H), 7.92–7.89 (m, 4H), 7.47 (t, *J* = 7.2 Hz, 2H), 7.31–7.27 (m, 3H), 7.19 (d, *J* = 6.8 Hz, 2H), 7.00–6.96 (m, 10H), 6.89–6.87 (m, 7H), 2.56 (t, *J* = 7.6 Hz, 8H), 1.60–1.55 (m, 8H), 1.34–1.30 (m, 24H), 0.87 (t, *J* = 6.4 Hz, 12H). ^13^C NMR (100 MHz, THF‐*d*_8_, δ): 145.24, 143.26, 143.01, 141.45, 133.77, 133.14, 132.76, 131.85, 130.92, 129.21, 128.58, 128.20, 128.09, 127.95, 127.63, 127.44, 125.81, 125.39, 60.79, 36.15, 32.49, 32.06, 29.91, 23.27, 14.20. HR‐MS (MALDI‐TOF) *m*/*z* calcd. for (C_84_H_86_): 1094.67295. Found: 1094.67069. Anal. Calcd. for C_84_H_86_: C, 92.09%; H, 7.91%; Found: C, 92.10%; H, 7.92%.

*N,N‐Bis(4‐(Hexyloxy)Phenyl)‐9,9,19,19‐Tetrakis(4‐Hexylphenyl)‐9,19‐Dihydrodinaphtho[3,2,1‐de:3′,2′,1′‐op]Pentacen‐5‐Amine (* ***7*** *)*: In a three‐neck round bottom flask, **4** (1.00 g, 0.91 mmol) was dissolved in chloroform (30 mL). NBS (162 mg, 0.91 mmol) was added to the reaction mixture, which was stirred at room temperature for 10 min. Then the organic phase was washed with plenty of water and dried over anhydrous sodium sulfate to yield a yellow solid as the intermediate product 5,15‐dibromo‐9,9,19,19‐tetrakis(4‐hexylphenyl)‐9,19‐dihydrodinaphtho[3,2,1‐*de*:3′,2′,1′‐*op*]pentacene, which was used for the next reaction directly. In a dried Schlenk tube were dissolved 5,15‐dibromo‐9,9,19,19‐tetrakis(4‐hexylphenyl)‐9,19‐dihydrodinaphtho[3,2,1‐*de*:3′,2′,1′‐*op*]pentacene (1.14 g, 0.91 mmol), **5** (337 mg, 0.91 mmol), and NaO*t*Bu (262 mg, 2.73 mmol) in toluene (15 mL). Then Pd_2_(dba)_3_ (33 mg, 0.036 mmol) and P(*t*‐Bu)_3_ (0.14 mL, 10 wt% in toluene, 0.055 mmol) were added to the reaction mixture in a nitrogen‐filled glove box, which was refluxed under argon for 12 h. The mixture was extracted three times with chloroform before the organic phase was washed with water and dried over anhydrous sodium sulfate. After solvent removal under reduced pressure, the crude product was purified by column chromatography (toluene/petroleum ether 60–90 °C, 1/3, *v*/*v*) on silica gel to yield a red solid as the desired product **7** (626 mg, 47% yield). ^1^H NMR (500 MHz, THF‐*d*_8_, δ): 8.36 (s, 1H), 8.09–7.92(m, 8H), 7.45–7.14(m, 9H), 7.01 (br, 10H), 6.89 (br, 10H), 6.68 (br, 4H), 3.84 (br, 4H), 2.57 (br, 8H), 1.60 (br, 6H), 1.30 (br, 42H), 0.87 (br, 18H). ^13^C NMR (125 MHz, THF‐*d*_8_, δ): 154.56, 145.28, 145.08, 143.43, 143.28, 143.25, 143.05, 142.77, 141.52, 141.48, 138.84, 133.75, 133.36, 133.26, 132.50, 131.79, 130.97, 130.33, 129.39, 129.18, 128.25, 128.03, 127.46, 126.29, 126.00, 125.86, 125.72, 125.44, 125.42, 124.08, 121.93, 115.66, 60.83, 36.16, 35.99, 32.68, 32.39, 32.09, 32.06, 30.54, 30.53, 30.45, 30.38, 30.36, 30.30, 30.15, 30.10, 30.08, 30.05, 29.93, 27.84, 27.83, 26.55, 26.23, 23.38, 23.34, 23.28, 14.24, 14.22. HR‐MS (MALDI‐TOF) *m*/*z* calcd. for (C_108_H_119_NO_2_): 1462.92744. Found: 1462.92076. Anal. Calcd. for C_108_H_119_NO_2_: C, 88.66%; H, 8.20%; N, 0.96%. Found: C, 88.67%; H, 8.21%; N, 0.95%.

*N,N‐Bis(2′,4′‐Bis(Hexyloxy)‐[1,1′‐Biphenyl]‐4‐yl)‐9,9,19,19‐Tetrakis(4‐Hexylphenyl)‐9,19‐Dihydrodinaphtho[3,2,1‐De:3′,2′,1′‐op]Pentacen‐5‐Amine (* ***8*** *)*: In a three‐neck round bottom flask, **4** (1.5 g, 1.37 mmol) was dissolved in chloroform (35 mL). NBS (488 mg, 2.74 mmol) was added to the reaction mixture, which was stirred at room temperature for 10 min. Then the organic phase was washed with plenty of water and dried over anhydrous sodium sulfate to yield a yellow solid as the intermediate product 5,15‐dibromo‐9,9,19,19‐tetrakis(4‐hexylphenyl)‐9,19‐dihydrodinaphtho[3,2,1‐*de*:3′,2′,1′‐*op*]pentacene, which was used for the next reaction directly. In a dried Schlenk tube were dissolved 5,15‐dibromo‐9,9,19,19‐tetrakis(4‐hexylphenyl)‐9,19‐dihydrodinaphtho[3,2,1‐*de*:3′,2′,1′‐*op*]pentacene (1.72 g, 1.37 mmol), **6** (2.48 g, 3.43 mmol), and NaO*t*Bu (395 mg, 4.11 mmol) in toluene (20 mL). Then Pd_2_(dba)_3_ (50 mg, 0.055 mmol) and P(*t*‐Bu)_3_ (0.21 mL, 10 wt% in toluene, 0.082 mmol) were added to the reaction mixture in a nitrogen‐filled glove box, which was refluxed under argon for 12 h. The mixture was extracted three times with chloroform before the organic phase was washed with water and dried over anhydrous sodium sulfate. After solvent removal under reduced pressure, the crude product was purified by column chromatography (toluene/petroleum ether 60–90 °C, 1/3, *v*/*v*) on silica gel to yield a red solid as the desired product **7** (1.04 g, 42% yield). ^1^H NMR (400 MHz, THF‐*d*_8_, δ): 8.36 (s, 1H), 8.18–8.16 (m, 2H), 8.12 (d, *J* = 7.0 Hz, 1H), 8.05–8.02 (m, 3H), 7.92 (t, *J* = 7.0 Hz, 2H), 7.45 (t, *J* = 5.8 Hz, 1H), 7.42–7.39 (m, 1H), 7.33 (d, *J* = 7.0 Hz, 4H), 7.28 (t, *J* = 5.6 Hz, 1H), 7.26–7.22 (m, 1H), 7.16–7.13 (m, 4H), 7.08–7.02 (m, 14H), 6.90 (br, 8H), 6.54–6.53 (m, 2H), 6.49–6.47 (m, 2H), 3.96–3.91 (m, 8H), 2.57 (br, 8H), 1.78–1.77 (m, 5H), 1.69–1.67 (m, 5H), 1.60 (br, 8H), 1.51–1.45 (m, 4H), 1.43–1.39 (m, 4H), 1.37–1.34 (m, 14H), 1.31–1.28 (m, 24H), 0.92 (t, *J* = 5.6 Hz, 6H), 0.87–0.82 (m, 18H). ^13^C NMR (125 MHz, THF‐*d*_8_, δ): 160.33, 157.75, 146.84, 143.51, 143.42, 143.08, 141.54, 141.49, 138.10, 133.76, 133.49, 133.24, 132.92, 132.56, 132.51, 131.81, 131.06, 130.95, 130.71, 129.31, 129.24, 129.17, 129.06, 128.57, 128.22, 127.95, 127.73, 127.48, 127.45, 126.55, 126.26, 125.81, 125.42, 125.33, 124.02, 123.83, 120.24, 105.97, 100.99, 68.80, 68.31, 67.74, 60.85, 36.16, 32.50, 32.41, 32.22, 32.11, 32.06, 30.12, 29.93, 29.86, 26.55, 26.46, 25.62, 23.35, 23.38, 23.24, 14.23, 14.21. HR‐MS (MALDI‐TOF) *m/z* calcd. for (C_132_H_151_NO_4_): 1815.16767. Found: 1815.16770. Anal. Calcd. for C_132_H_151_NO_4_: C, 87.32%; H, 8.38%; N, 0.77%. Found: C, 87.33%; H, 8.39%; N, 0.76%.

*4‐(7‐((15‐(Bis(4‐(Hexyloxy)Phenyl)Amino)‐9,9,19,19‐Tetrakis(4‐Hexylphenyl)‐9,19‐Dihydrodinaphtho[3,2,1‐de:3*′*,2*′*,1*′*‐op]Pentacen‐5‐yl)Ethynyl)Benzo[c][1,2,5]Thiadiazol‐4‐yl)Benzoic Acid (* ***R1*** *)*: In a three‐neck round bottom flask, **7** (626 mg, 0.43 mmol) was dissolved in THF (15 mL). NBS (80 mg, 0.45 mmol) was added to the reaction mixture, which was stirred at room temperature for 1 h. Chloroform was added before the organic phase was washed with water and dried over anhydrous sodium sulfate. After solvent removal under reduced pressure, the crude product was purified by column chromatography (toluene/petroleum ether 60–90 °C, 1/3, *v*/*v*) on silica gel to yield an orange–red solid as the intermediate product 15‐bromo‐*N*,*N*‐bis(4‐(hexyloxy)phenyl)‐9,9,19,19‐tetrakis(4‐hexylphenyl)‐9,19‐dihydrodinaphtho[3,2,1‐*de*:3′,2′,1′‐*op*]pentacen‐5‐amine, which was used for the next reaction directly. In a dried Schlenk tube were dissolved 15‐bromo‐*N*,*N*‐bis(4‐(hexyloxy)phenyl)‐9,9,19,19‐tetrakis(4‐hexylphenyl)‐9,19‐dihydrodinaphtho[3,2,1‐*de*:3′,2′,1′‐*op*]pentacen‐5‐amine (663 mg, 0.43 mmol), butyl 4‐(7‐ethynylbenzo[c][1,2,5]thiadiazol‐4‐yl)benzoate (289 mg, 0.86 mmol), and Cs_2_CO_3_ (154 mg, 0.47 mmol) in dioxane (20 mL). Then Pd_2_(dba)_3_ (24 mg, 0.026 mmol) and P(*t*‐Bu)_3_ (0.13 mL, 10 wt% in toluene, 0.052 mmol) were added to the reaction mixture in a nitrogen‐filled glove box, which was refluxed under argon for 12 h. The mixture was extracted three times with chloroform before the organic phase was washed with water and dried over anhydrous sodium sulfate. After solvent removal under reduced pressure, the crude product was purified by column chromatography (toluene/petroleum ether 60–90 °C, 1/1, *v*/*v*) on silica gel to yield a purple powder as the desired butyl ester. In a three‐neck round‐bottom flask were dissolved the above butyl ester and KOH (241 mg, 4.30 mmol) in a solvent mixture of THF/H_2_O (20 mL, 3/1, *v*/*v*). The reaction mixture was refluxed for 9 h and then cooled to room temperature. Chloroform was added before the organic phase was washed with 0.1 m phosphoric acid and deionized water in turn and then dried over anhydrous sodium sulfate. After solvent removal under reduced pressure, the crude product was purified by column chromatography (chloroform/methanol, 20/1, *v*/*v*) on silica gel to yield a purple powder as the desired product **R1** (614 mg, 82% yield). ^1^H NMR (500 MHz, THF‐*d*_8_, δ): 8.95 (d, *J* = 9.2 Hz, 1H), 8.91 (d, *J* = 8.5 Hz, 1H), 8.21–8.17 (m, 4H), 8.13–8.08 (m, 4H), 8.05 (d, *J* = 8.8 Hz, 1H), 8.00 (d, *J* = 5.6 Hz, 2H), 7.92 (d, *J* = 7.4 Hz, 1H), 7.64 (t, *J* = 7.1 Hz, 1H), 7.48 (t, *J* = 6.9 Hz, 1H), 7.37 (t, *J* = 8.6 Hz, 1H), 7.24–7.14 (m, 4H), 7.10–7.03 (m, 11H), 6.93–6.91 (m, 10H), 6.70–6.67 (m, 4H), 3.84 (t, *J* = 6.5 Hz, 4H), 2.58–2.57 (m, 8H), 1.70–1.68 (m, 2H), 1.59 (br, 8H), 1.47–1.41 (m, 4H), 1.34–1.30(m, 34H), 0.91–0.86 (m, 18H). ^13^C NMR (125 MHz, THF‐*d*_8_, δ): 167.21, 156.40, 154.61, 153.78, 143.83, 143.45, 142.78, 141.73, 141.68, 141.62, 139.21, 134.67, 134.03, 133.75, 133.21, 133.06, 132.69, 132.50, 132.39, 131.51, 130.97, 130.92, 130.42, 130.34, 130.32, 129.85, 129.52, 129.31, 128.97, 128.45, 128.33, 128.09, 127.76, 127.72, 127.60, 127.09, 126.83, 126.35, 126.23, 126.06, 125.89, 124.18, 121.98, 117.78, 117.46, 115.68, 99.07, 95.16, 68.47, 67.74, 60.95, 60.82, 36.16, 35.99, 32.68, 32.39, 32.09, 32.07, 30.54, 30.52, 30.45, 30.36, 30.29, 30.16, 30.10, 30.07, 30.05, 29.94, 29.92, 27.82, 26.55, 26.22, 25.62, 23.38, 23.34, 23.29, 23.28, 14.26, 14.23, 14.22, 14.21. HR‐MS (MALDI‐TOF) *m/z* calcd. for (C_123_H_125_N_3_O_4_S): 1740.94244. Found: 1740.93722. Anal. Calcd. for C_123_H_125_N_3_O_4_S: C, 84.84%; H, 7.24%; N, 2.41%. Found: C, 84.83%; H, 7.25%; N, 2.40%.

*4‐(7‐((15‐(Bis(2′,4′‐Bis(Hexyloxy)‐[1,1′‐Biphenyl]‐4‐yl)Amino)‐9,9,19,19‐Tetrakis(4‐Hexylphenyl)‐9,19‐Dihydrodinaphtho[3,2,1‐de:3′,2′,1′‐op]Pentacen‐5‐yl)Ethynyl)Benzo[c][1,2,5]Thiadiazol‐4‐yl)Benzoic Acid (* ***R2*** *)*: In a three‐neck round bottom flask, **8** (830 mg, 0.46 mmol) was dissolved in THF (30 mL). NBS (82 mg, 0.46 mmol) was added to the reaction mixture, which was stirred at room temperature for 1 h. Chloroform was added before the organic phase was washed with water and dried over anhydrous sodium sulfate. After solvent removal under reduced pressure, the crude product was purified by column chromatography (toluene/petroleum ether 60–90 °C, 1/3, *v*/*v*) on silica gel to yield an orange–red solid as the intermediate product *N*,*N*‐bis(2′,4′‐bis(hexyloxy)‐[1,1′‐biphenyl]‐4‐yl)‐15‐bromo‐9,9,19,19‐tetrakis(4‐hexylphenyl)‐9,19‐dihydrodinaphtho[3,2,1‐*de*:3′,2′,1′‐*op*]pentacen‐5‐amine. In a dried Schlenk tube were dissolved 15‐bromo‐*N*,*N*‐bis(4‐(hexyloxy)phenyl)‐9,9,19,19‐tetrakis(4‐hexylphenyl)‐9,19‐dihydrodinaphtho[3,2,1‐*de*:3′,2′,1′‐*op*]pentacen‐5‐amine (872 mg, 0.46 mmol), butyl 4‐(7‐ethynylbenzo[c][1,2,5]thiadiazol‐4‐yl)benzoate (464 mg, 1.38 mmol), and Cs_2_CO_3_ (163 mg, 0.51 mmol) in dioxane (20 mL). Then Pd_2_(dba)_3_ (26 mg, 0.028 mmol) and P(*t*‐Bu)_3_ (0.14 mL, 10 wt% in toluene, 0.055 mmol) were added to the reaction mixture in a nitrogen‐filled glove box, which was refluxed under argon for 12 h. The mixture was extracted three times with chloroform before the organic phase was washed with water and dried over anhydrous sodium sulfate. After solvent removal under reduced pressure, the crude product was purified by column chromatography (toluene/petroleum ether 60–90 °C, 1/2, *v*/*v*) on silica gel to yield a violet powder as the desired butyl ester. In a three‐neck round‐bottom flask were dissolved the above butyl ester and KOH (258 mg, 4.60 mmol) in a solvent mixture of THF/H_2_O (20 mL, 3/1, *v*/*v*). The reaction mixture was refluxed for 9 h and then cooled to room temperature. Chloroform was added before the organic phase was washed with 0.1 m phosphoric acid and deionized water in turn and then dried over anhydrous sodium sulfate. After solvent removal under reduced pressure, the crude product was purified by column chromatography (chloroform/methanol, 20/1, *v*/*v*) on silica gel to yield a purple powder as the desired product **R2** (818 mg, 85% yield). ^1^H NMR (500 MHz, THF‐*d*_8_, δ): 8.95–8.91(m, 2H), 8.21–8.15(m, 7H), 8.11 (d, *J* = 7.4 Hz, 1H), 8.08–8.06 (m, 2H), 8.02 (s, 1H), 7.93 (d, *J* = 7.4 Hz, 1H), 7.65 (t, *J* = 7.1 Hz, 1H), 7.50 (t, *J* = 6.9 Hz, 1H), 7.44–7.41 (m, 1H), 7.35–7.33 (m, 4H), 7.27–7.24 (m, 2H), 7.18–7.15 (m, 4H), 7.12–7.04 (m, 14H), 7.09 (br, 7H), 6.54–6.53 (m, 2H), 6.49–6.47 (m, 2H), 3.96–3.92 (m, 8H), 2.58 (br, 8H), 1.59 (br, 8H), 1.51–1.45 (m, 6H), 1.34–1.28 (m, 50H), 0.93–0.82 (m, 24H). ^13^C NMR (125 MHz, THF‐*d*_8_, δ): 167.33, 160.33, 157.74, 156.41, 153.79, 146.84, 144.84, 144.81, 143.87, 143.59, 143.57, 143.35, 141.69, 141.64, 138.46, 134.67, 134.02, 133.78, 133.36, 133.18, 133.05, 132.73, 132.56, 132.48, 131.64, 131.07, 130.90, 130.74, 130.42, 130.34, 130.32, 129.83, 129.43, 129.29, 129.00, 128.85, 128.51, 128.45, 128.34, 128.11, 127.77, 127.63, 127.11, 126.84, 126.64, 126.34, 126.26, 125.99, 125.41, 124.12, 123.77, 120.28, 117.75, 117.50, 105.96, 100.97, 99.07, 95.14, 68.79, 68.30, 67.74, 60.95, 60.83, 36.16, 36.60, 32.68, 32.49, 32.40, 32.22, 32.10, 32.06, 30.54, 30.52, 30.45, 30.37, 30.35, 30.29, 30.15, 30.05, 29.94, 29.85, 27.83, 26.55, 26.46, 26.23, 25.62, 25.54, 23.35, 23.28, 23.25, 14.24, 14.22, 1.21. HR‐MS (MALDI‐TOF) *m*/*z* calcd. for (C_147_H_157_N_3_O_6_S): 2093.18267. Found: 2093.18111. Anal. Calcd. for C_147_H_157_N_3_O_6_S: C, 84.32%; H, 7.56%; N, 2.01%. Found: C, 84.33%; H, 7.55%; N, 2.02%.

*Theoretical Calculations*: The 6–311G(d,p) basis set was applied for all theoretical calculations with Gaussian 09. The optimization of ground state geometries was carried out using the popular B3LYP exchange‐correlation functional.^20^ The TD‐MPW1K hybrid functional was picked to calculate the vertical excitation energies.^21^, ^22^ The solvent effect on the geometries and transition energies were taken into account by means of the conductor‐like polarizable continuum model (CPCM).^23^

*Electrochemical and Photophysical Measurements*: Cyclic voltammograms (CVs) of dye molecules in THF were recorded on a CHI660C electrochemical workstation to derive the HOMO/LUMO energy levels.^24^ A three‐electrode electrolytic cell was used and all potentials were reported with Fc/Fc^+^ as the internal reference. Static electronic absorption spectra were measured with an Agilent G1103A spectrometer equipped with a silicon diode array detector. Nanosecond laser flash photolysis measurements were performed with an LP920 laser flash spectrometer. The probe light at 785 nm generated from an LDM 785 laser diode module (Thorlabs Corp.) was detected by a silicon detector in connection with a TDS 3012C digital signal analyzer. Time‐correlated single‐photon counting measurements were carried out with a LifeSpec‐II spectrometer, employing an EPL485 pulsed laser diode and a MCP‐PMT detector. The details of femtosecond fluorescence up‐conversion measurements were outlined in our previous paper.^25^

*Cell Fabrication and Characterization*: A 4.5+5 µm thick, double layer titania film screen‐printed on a precleaned Ffuorine‐doped tin oxide (FTO) conducting glass (NSG, Solar) was employed as the negative electrode of DSCs and further dye‐loaded by immersing it into a dye solution made by dissolving 150 µm of dye in a chloroform‐ethanol mixture (*v*/*v*, 3/7) for 12 h. The details for film preparation were described in a previous paper.^26^ The dye‐coated titania electrode was assembled with a thermally platinized FTO electrode by using a 25‐µm‐thick Surlyn ring to produce a thin‐layer electrochemical cell. The Co‐bpy electrolyte was made from 0.25 m tris(2,2′‐bipyridine)cobalt(II) di[bis(trifluoromethanesulfonyl)imide], 0.05 m tris(2,2′‐bipyridine)cobalt(III) tris[bis(trifluoromethanesulfonyl)imide], 0.5 m TBP, and 0.1 m LiTFSI in acetonitrile. Detailed description on photovoltaic characterization such as EQE, *J*–*V*, CE, and TPD measurements have been described in our previous publications.

## Conflict of Interest

The authors declare no conflict of interest.

## Supporting information

Supplementary
