# Supplementary material for: 2H‐Dinaphthopentacene: A Polycyclic Aromatic Hydrocarbon Core for Metal‐Free Organic Sensitizers in Efficient Dye‐Sensitized Solar Cells
Source: Adv Sci (Weinh). 2017 Apr 25;4(9):1700099. doi: 10.1002/advs.201700099 (PMC5604383; doi:10.1002/advs.201700099)
Supplement: Supplementary file 1 — Supplementary [file ADVS-4-na-s001.pdf]

Copyright WILEY-VCH Verlag GmbH & Co. KGaA, 69469 Weinheim, Germany, 2017.

ADVANCED SCIENCE Open Access

Supporting Information

for *Adv. Sci.,* DOI: 10.1002/advs.201700099

2*H*-Dinaphthopentacene: A Polycyclic Aromatic Hydrocarbon Core for Metal-Free Organic Sensitizers in Efficient Dye-Sensitized Solar Cells

*Yameng Ren, Jiao Liu, Aibin Zheng, Xiandui Dong, and Peng Wang**

Copyright WILEY-VCH Verlag GmbH & Co. KGaA, 69469 Weinheim, Germany, 2016.

Supporting Information

2*H*-Dinaphthopentacene: a Polycyclic Aromatic Hydrocarbon Core for Metal-Free Organic Sensitizers in Efficient Dye-Sensitized Solar Cells

*Yameng Ren, Jiao Liu, Aibin Zheng, Xiandui Dong, and Peng Wang**

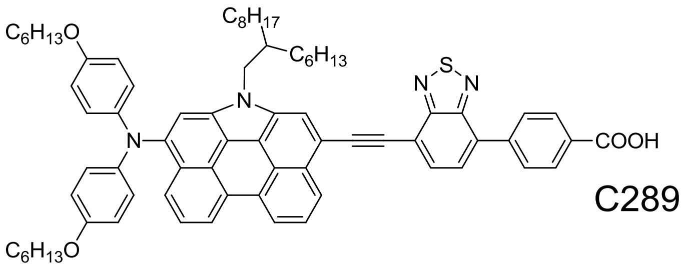

C289

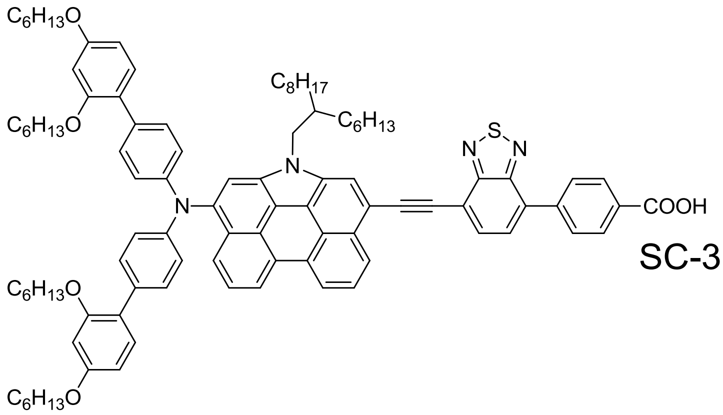

SC-3

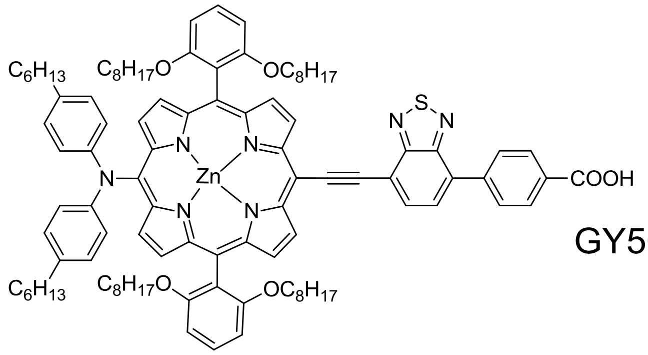

GY50

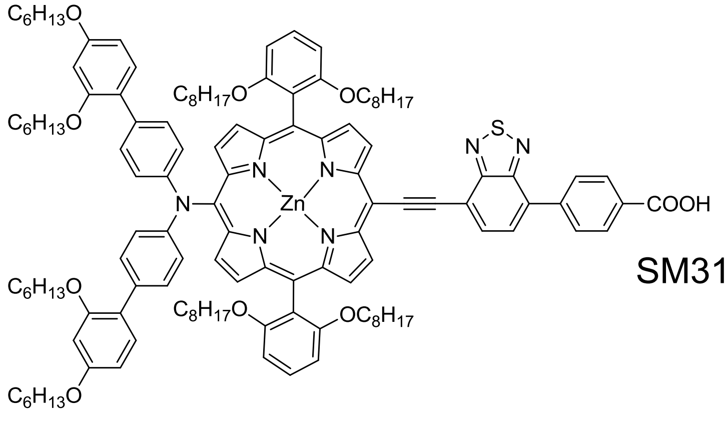

SM315

Figure S1. Molecular structures of C289, SC-3, GY50, and SM315.

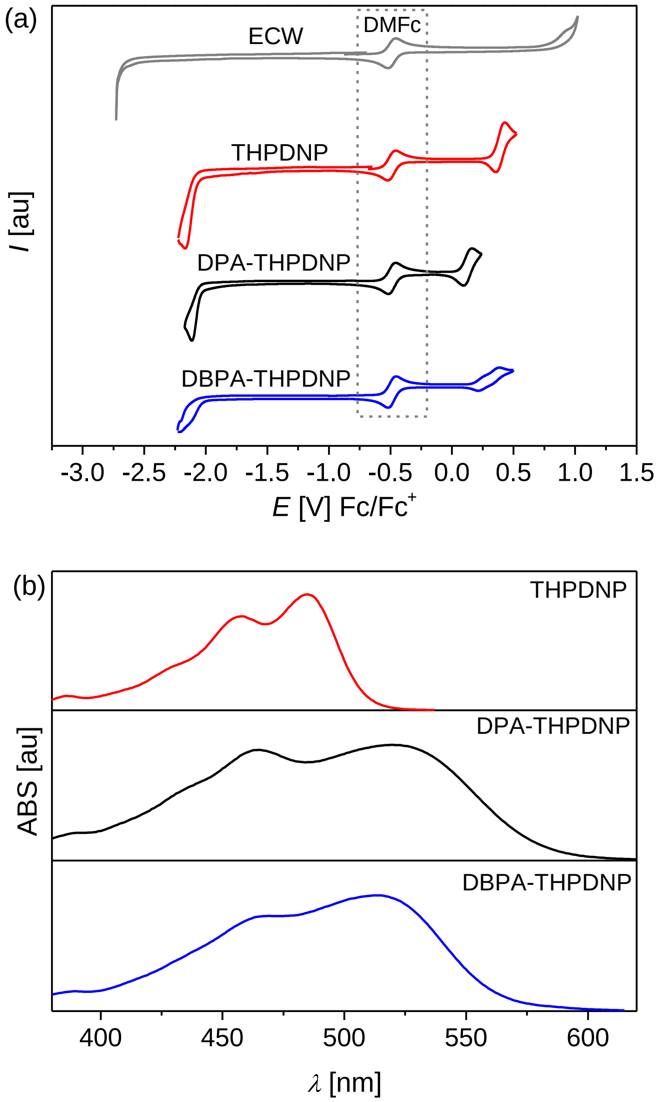

Figure S2. (a) Cyclic voltammograms of THPDNP, DPA-THPDNP, and DBPA-THPDNP in THF using 1-ethyl-3-methylimidazolium bis(trifluoromethanesulfonyl)imide (EMITFSI) as the supporting electrolyte. Scan rate: 5 mV s^{-1}. Decamethylferrocene (DMFc) was added as the internal reference and all potentials were further calibrated with the standard redox couple ferrocene/ferrocenium ($Fc_/Fc^+$). Electrochemical window (ECW) with a glassy carbon working electrode is also included. (b) Steady-state UV-vis spectroscopies of THPDNP, DPA-THPDNP, and DBPA-THPDNP in THF.

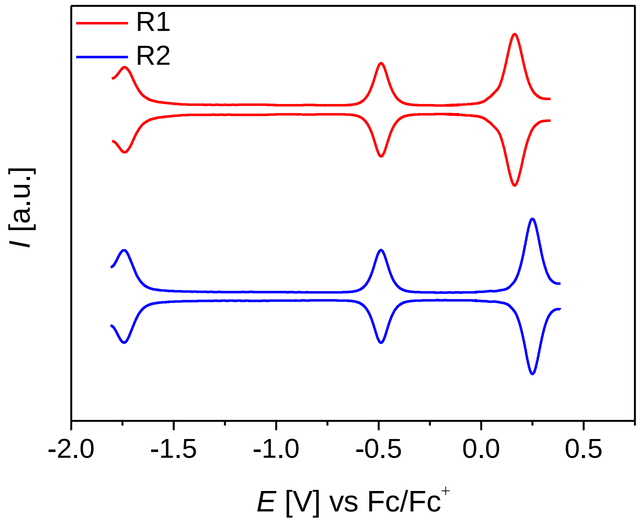

Figure S3. Square-wave voltammograms of dyes in THF with 0.1 M EMITFSI as supporting electrolyte. DMFc was added as the internal reference and all potentials were further calibrated with the standard redox couple Fc/Fc^{+}.

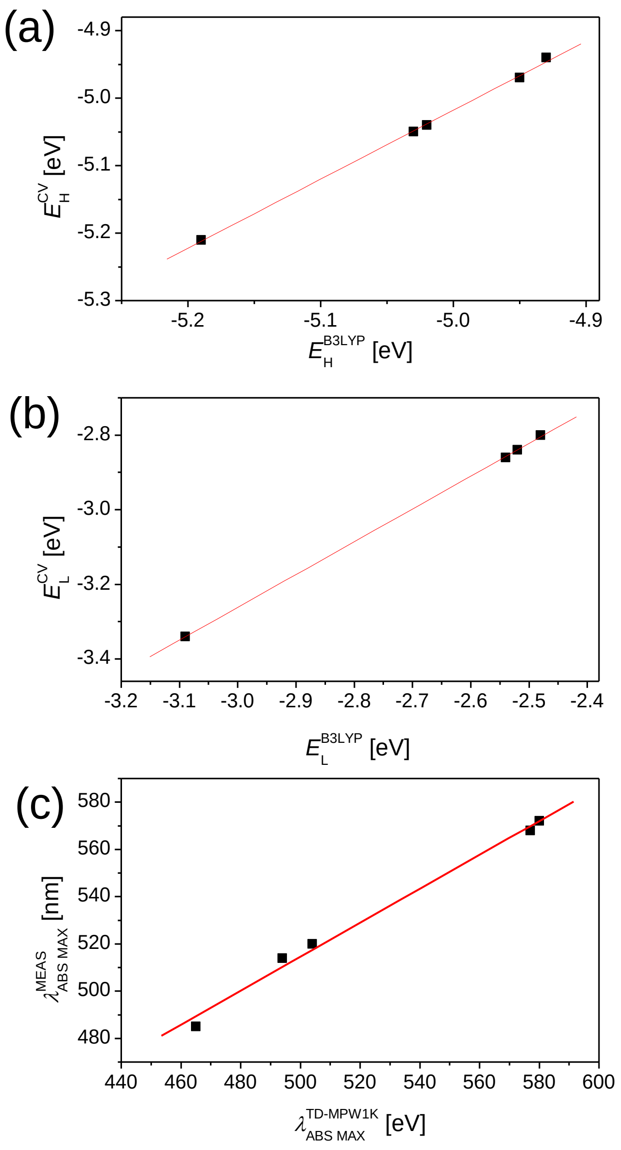

Figure S4. Scatter plots of experimentally measured and theoretically calculated physical parameters: (a) $E_{\mathrm{H}}^{\mathrm{CV}}$ vs. $E_{\mathrm{H}}^{\mathrm{B3LYP}}$, (b) $E_{\mathrm{L}}^{\mathrm{CV}}$ vs. $E_{\mathrm{L}}^{\mathrm{B3LYP}}$, and (c) $\lambda_{\mathrm{ABS\ MAX}}^{\mathrm{MEAS}}$ vs. $\lambda_{\mathrm{ABS\ MAX}}^{\mathrm{TD\text{-}MPW1K}}$. The red solid lines are linear fittings.

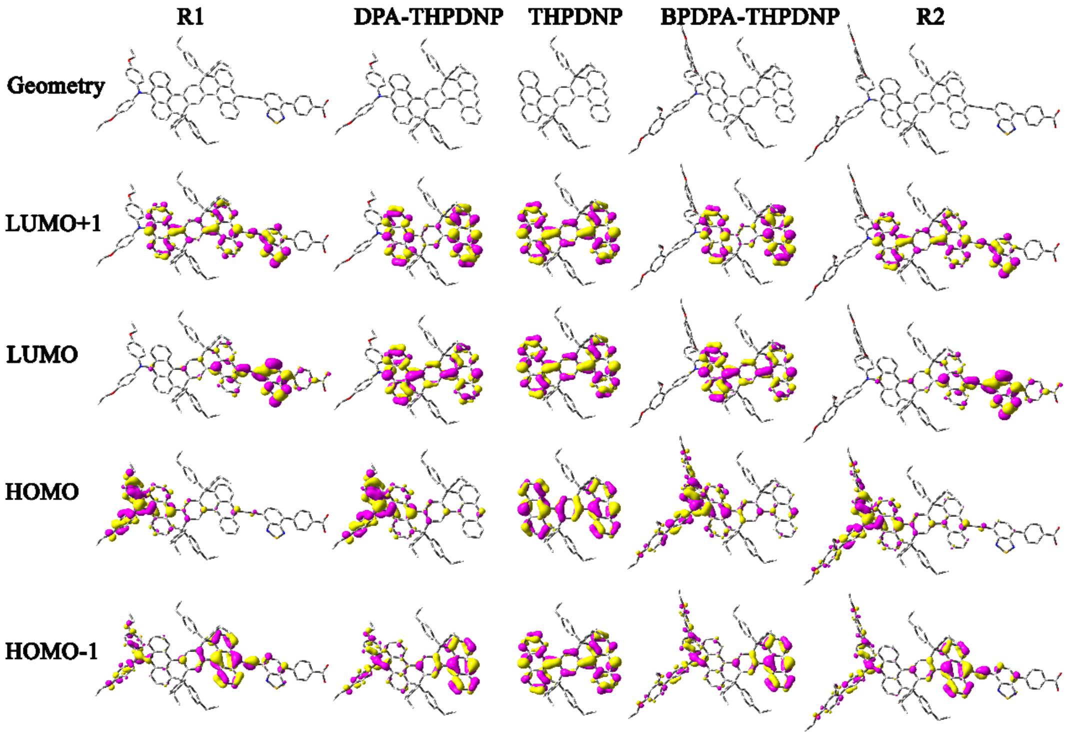

Figure S5. Optimized geometries and contour plots of frontier molecular orbitals at the B3LYP/6-311G(d,p) level of dye molecules in THF and their corresponding electron donors. The large aliphatic substituents were cut to ethyl for improved computational efficiencies.

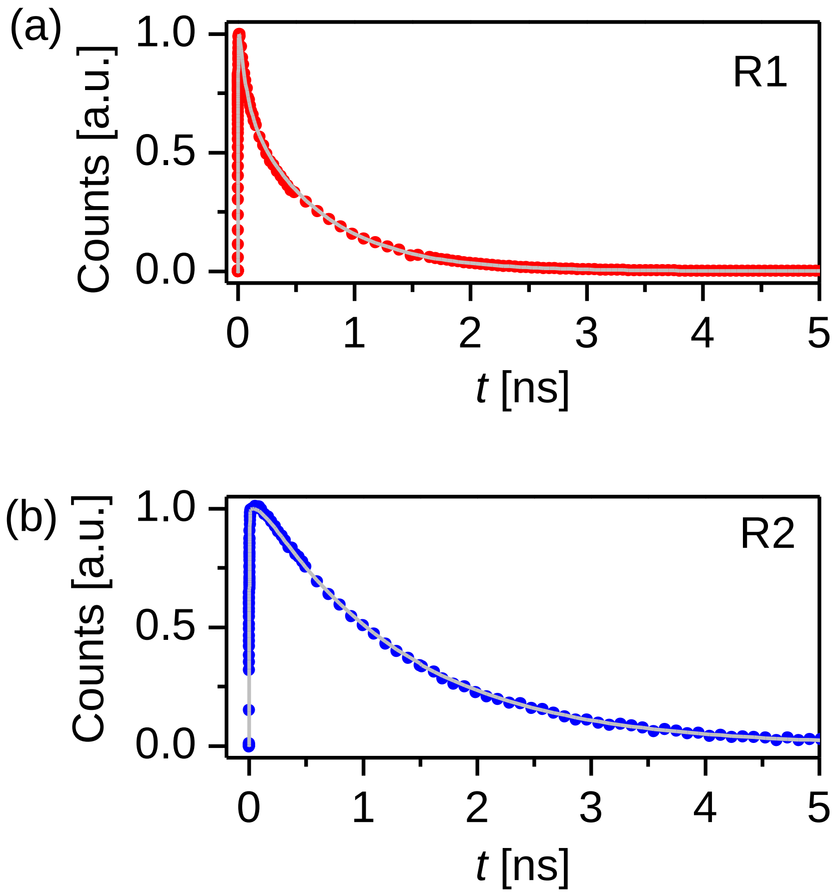

Figure S6. Up-converted photoluminescence traces of **R1** and **R2** in THF probed at 850 nm. The gray fitting lines are also included. Pump wavelength: 490 nm; pulse fluence: 28 μJ cm^{-2}.

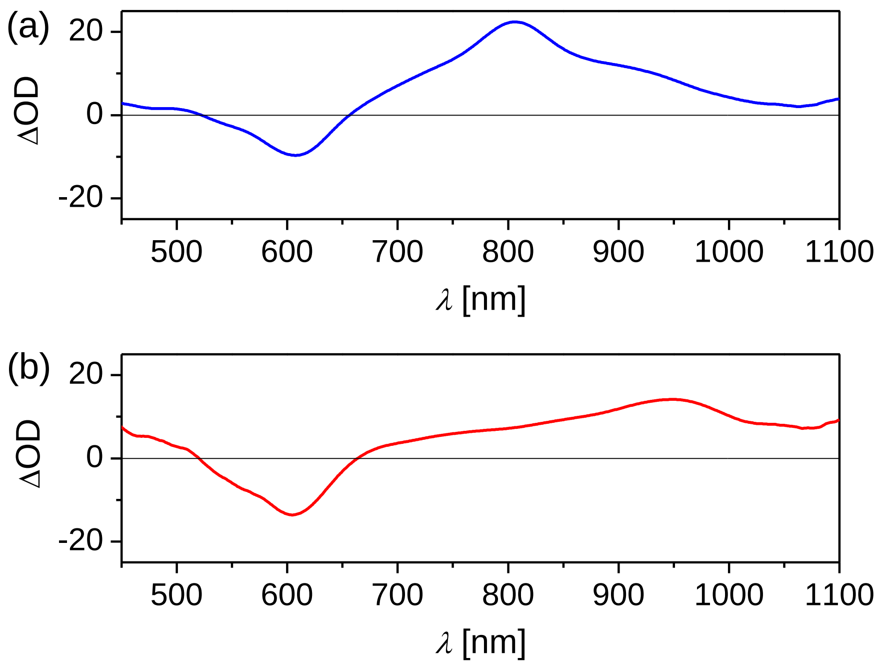

Figure S7. Absorption change of a 1.2-μm-thick, dye-grafted titania film immersed in EMITFSI after applying a positive potential bias: (a) **R1** and (b) **R2**.

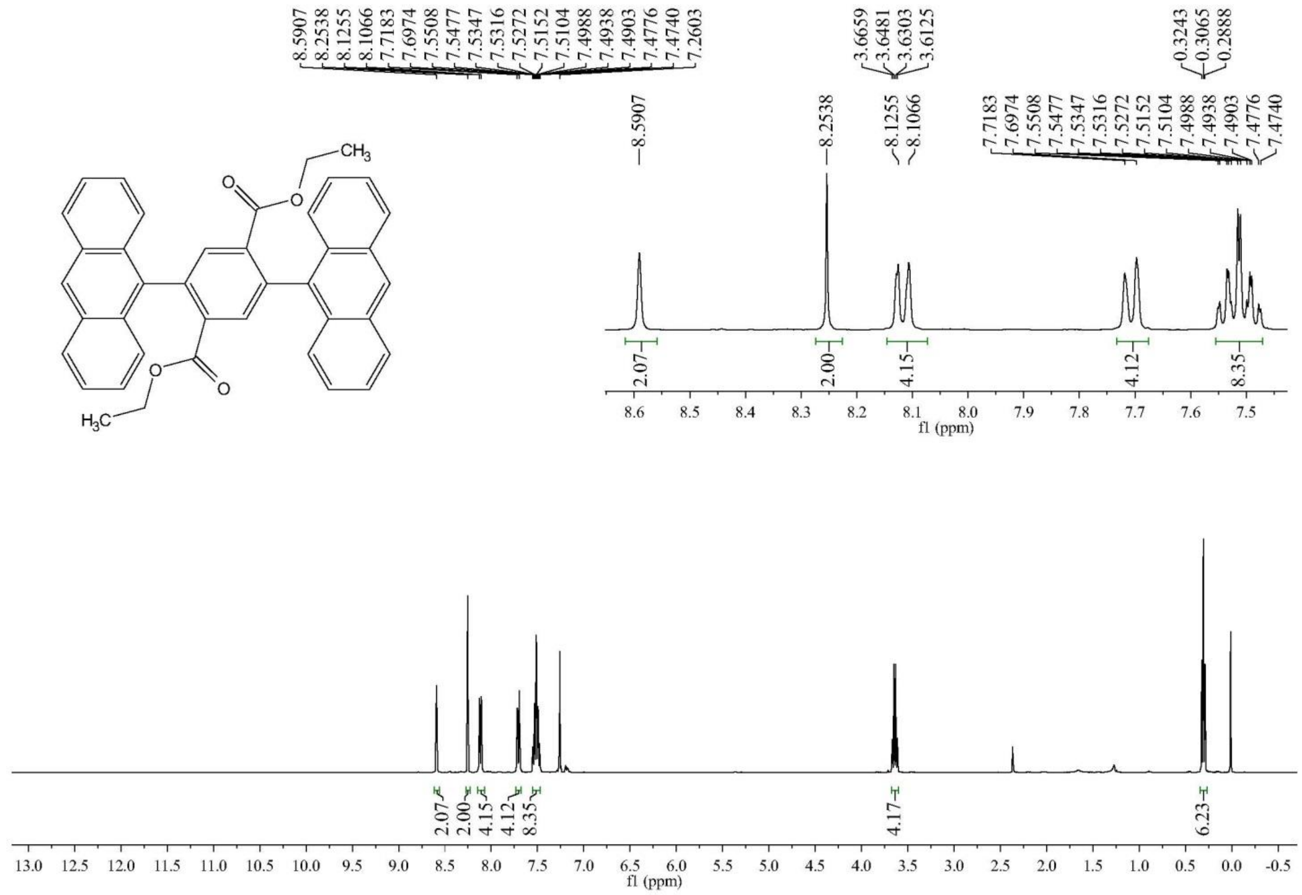

Figure S8. The ^{1}H NMR (400 MHz) spectrum of **3** in $CDCl_3$.

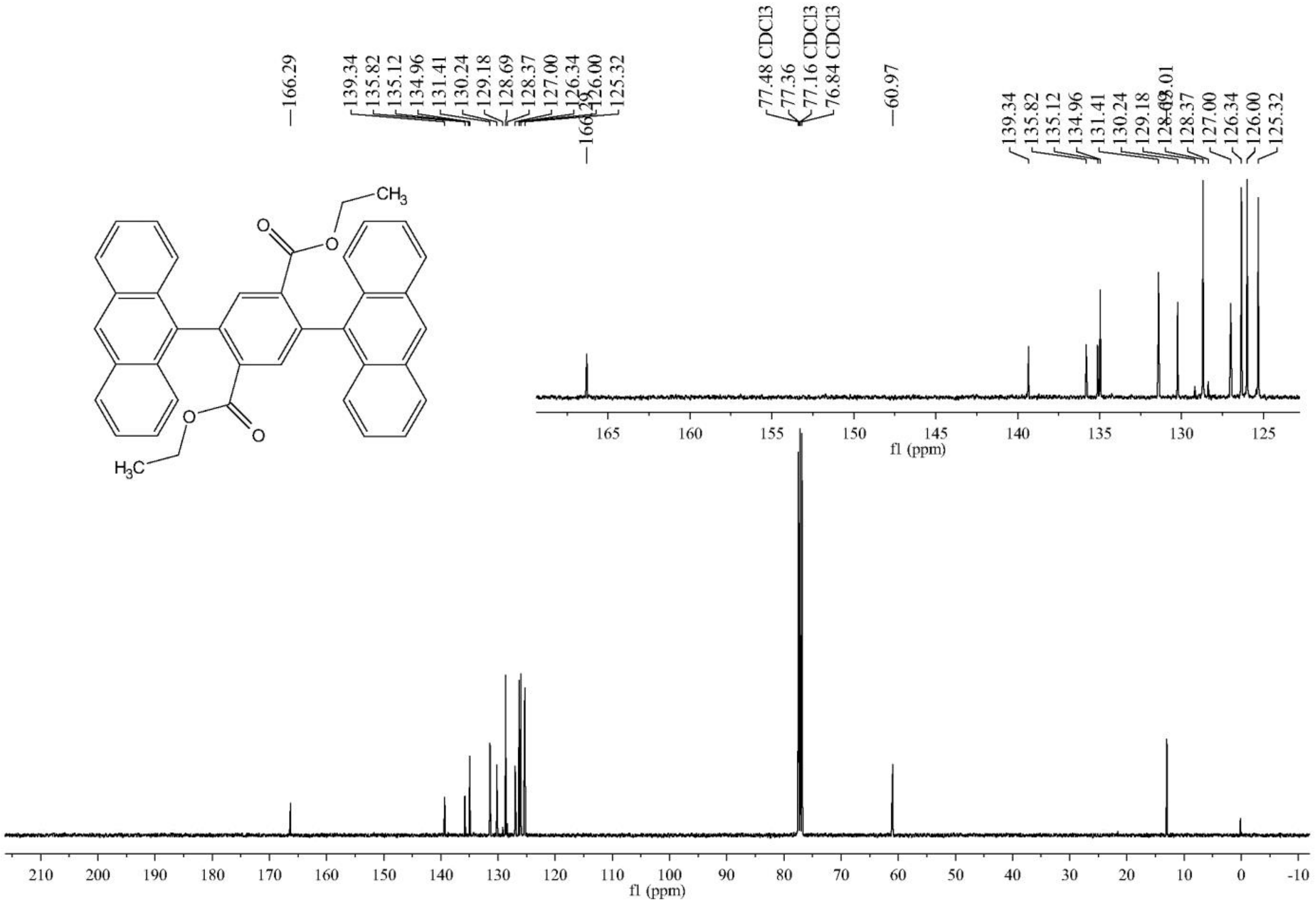

Figure S9. The ^{13}C NMR (100 MHz) spectrum of **3** in $CDCl_3$.

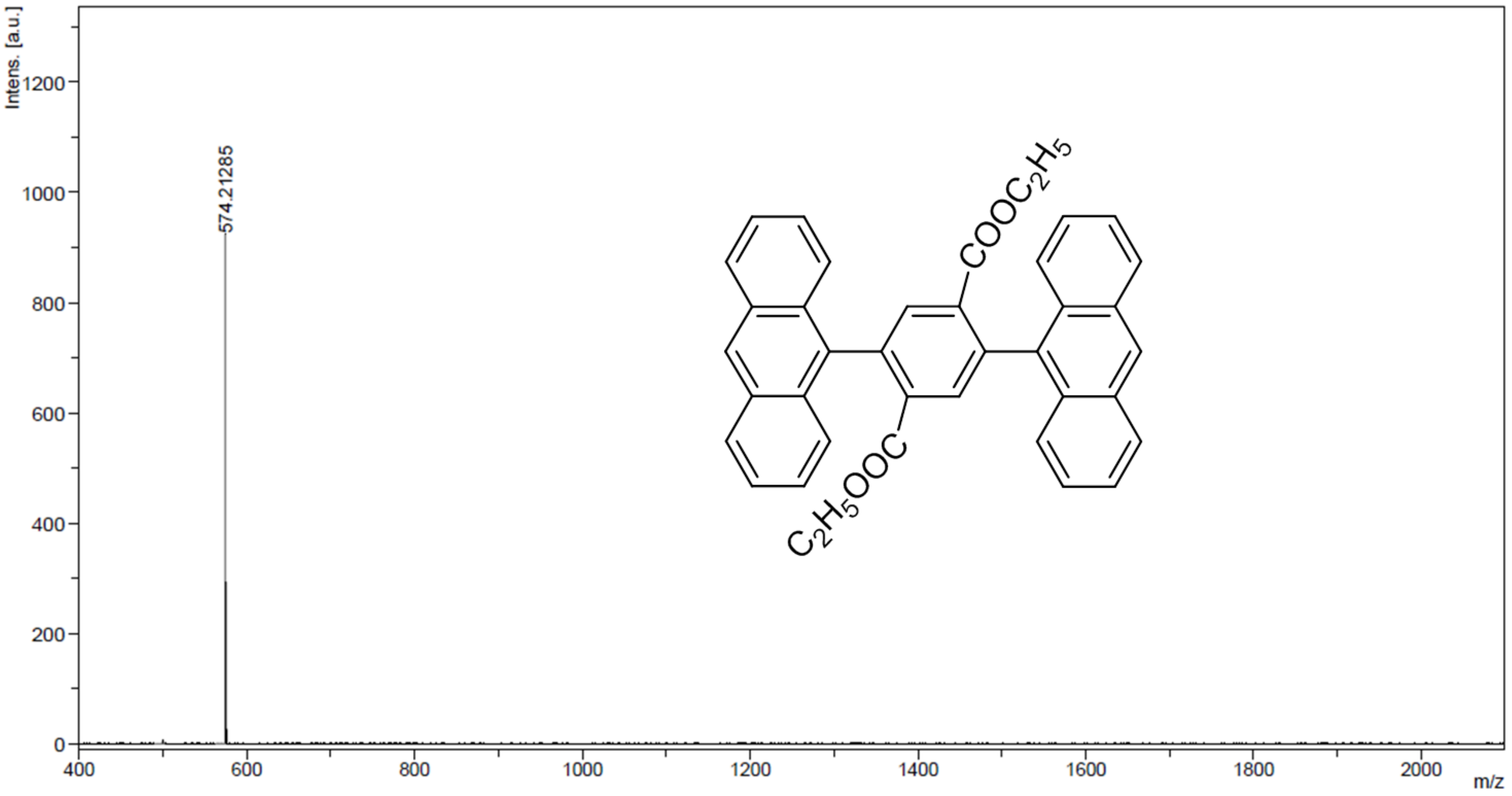

Figure S10. The high resolution mass spectrum (MALDI-TOF) of **3**.

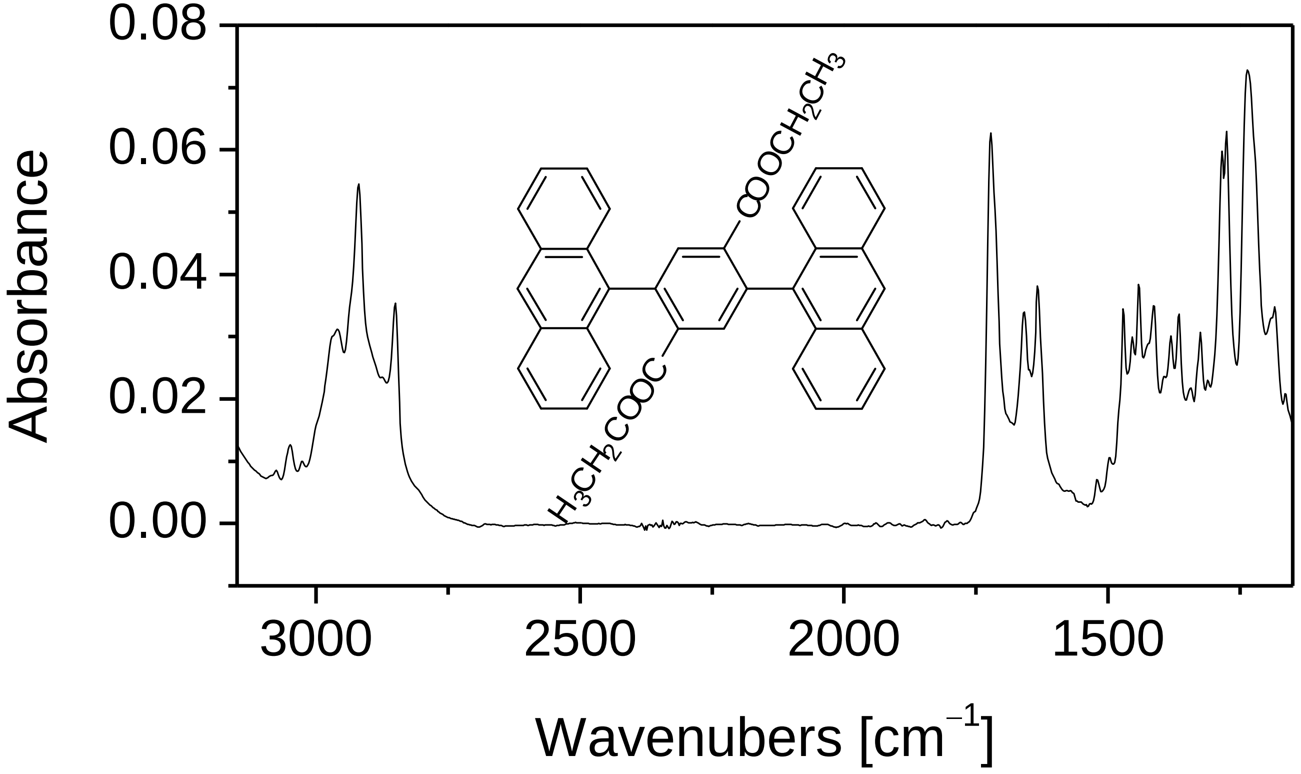

Figure S11. The ATR-FTIR spectrum of **3**.

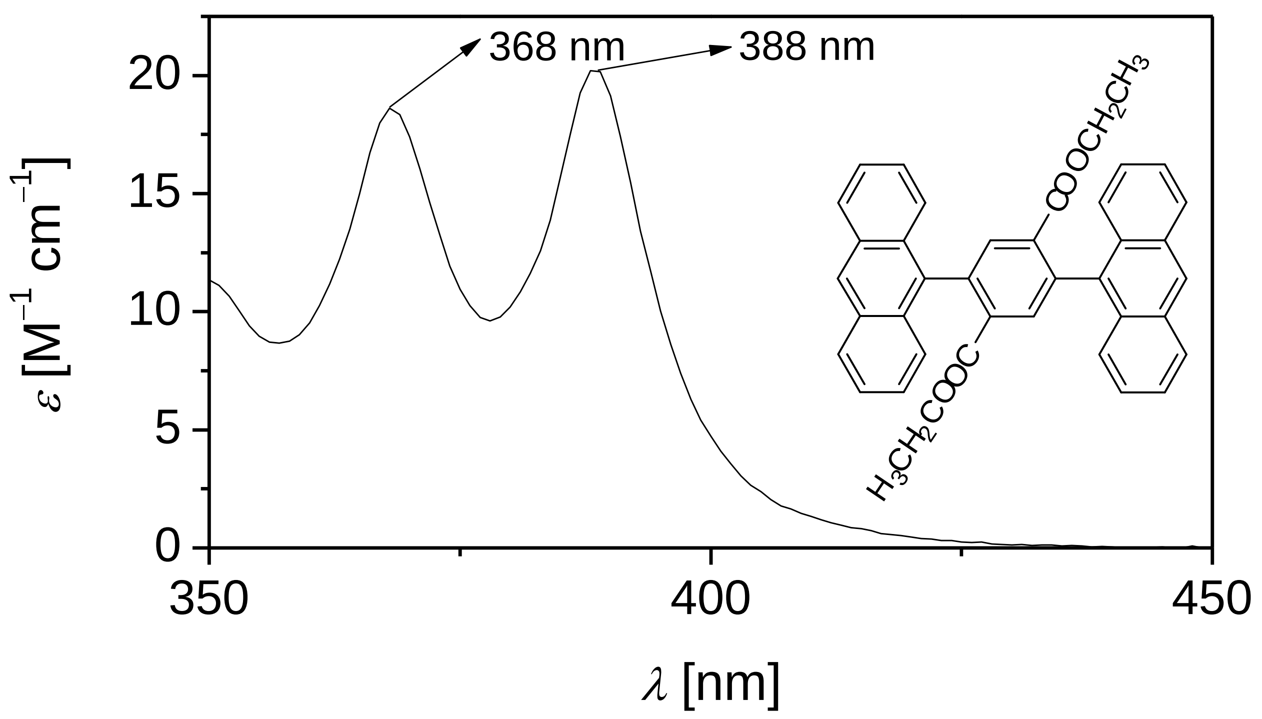

Figure S12. The UV-Vis spectroscopy of **3** in THF.

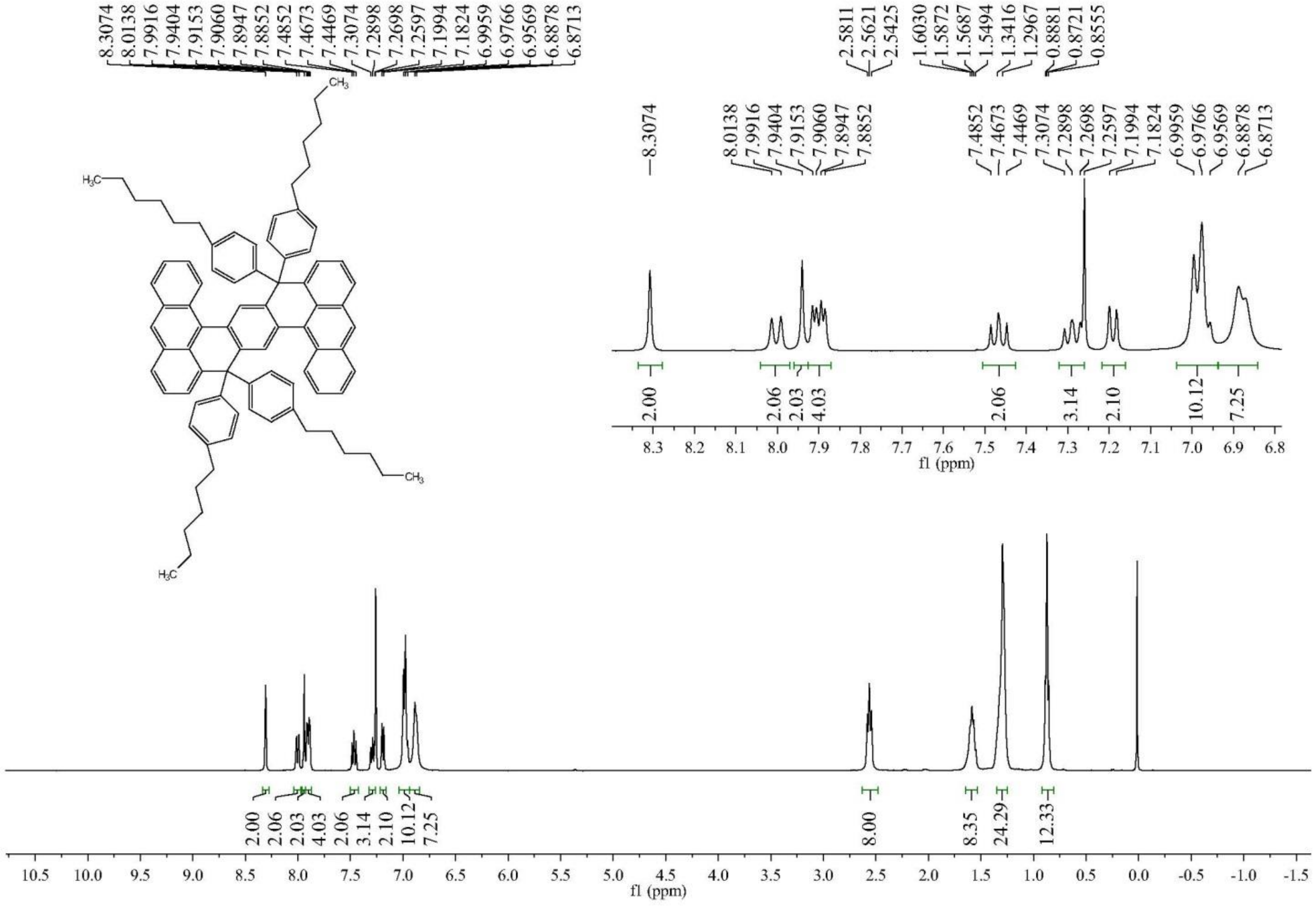

Figure S13. The ^{1}H NMR (400 MHz) spectrum of **4** in $CDCl_3$.

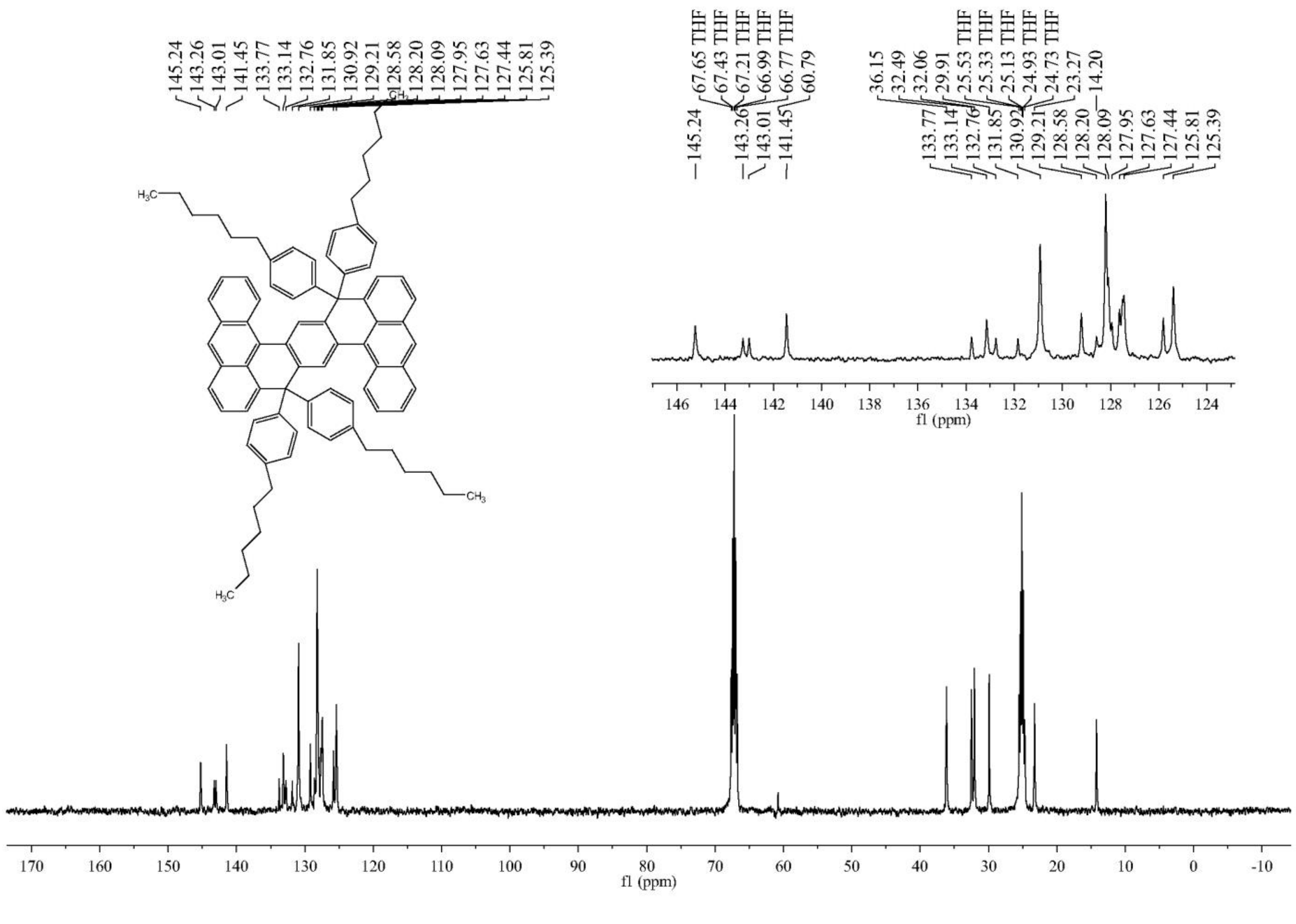

Figure S14. The ^{13}C NMR (100 MHz) spectrum of **4** in THF-d_8.

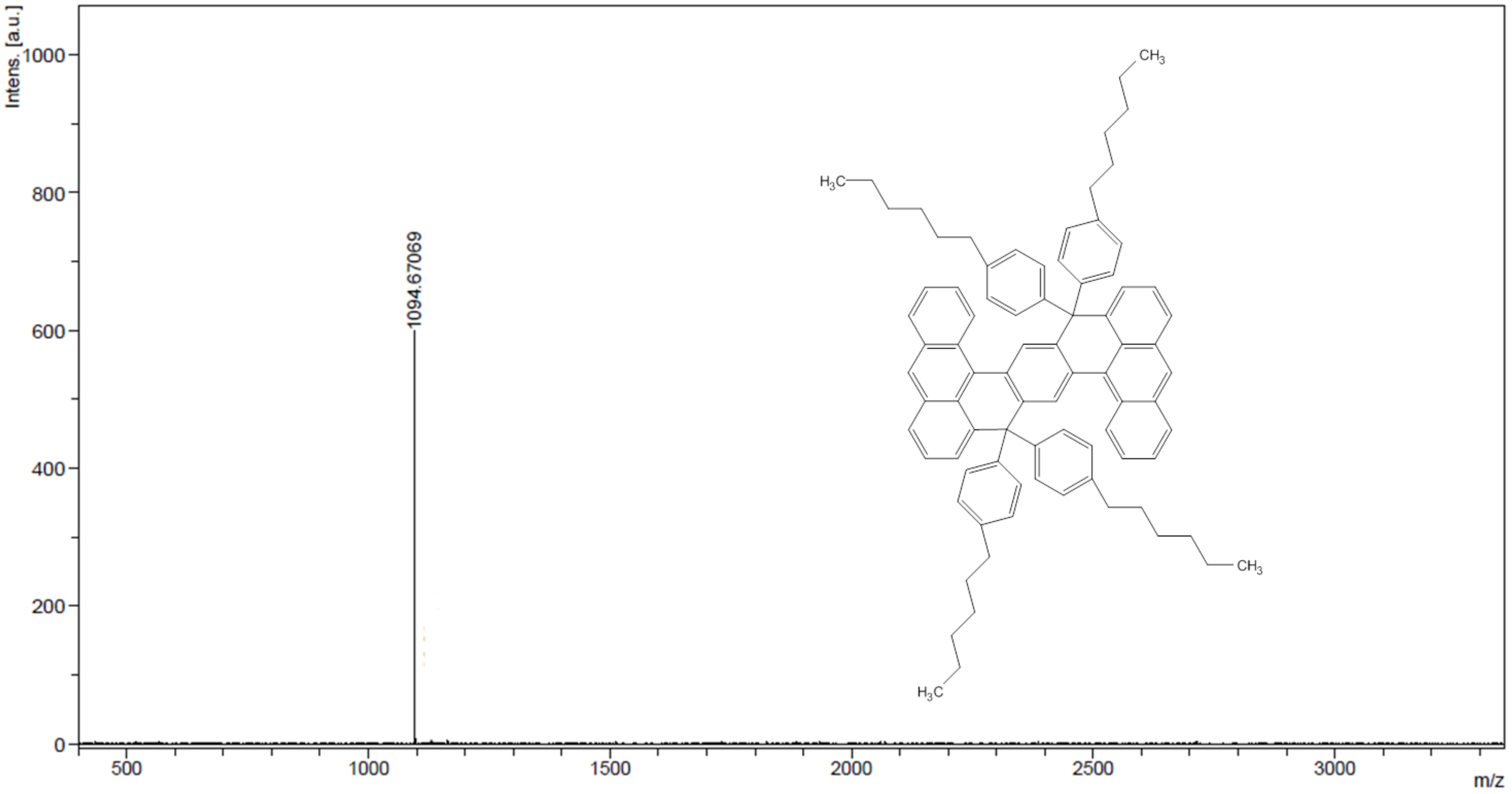

Figure S15. The high resolution mass spectrum (MALDI-TOF) of **4**.

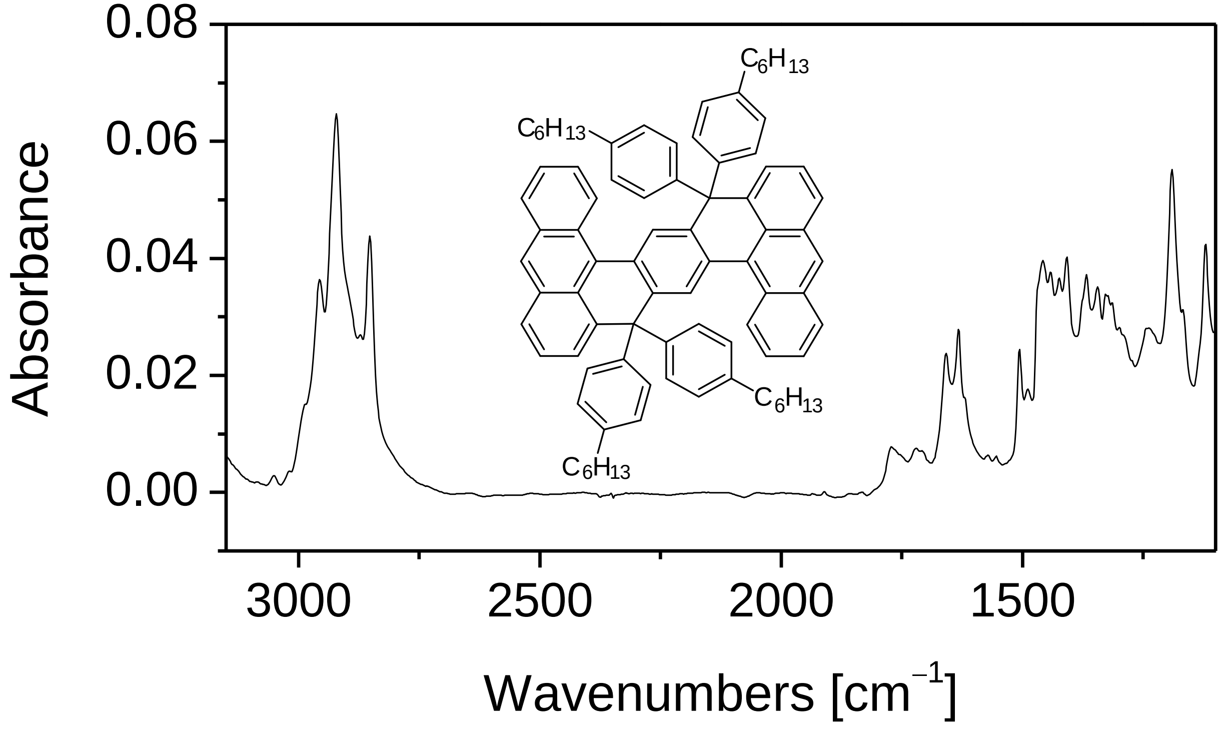

Figure S16. The ATR-FTIR spectrum of **4**.

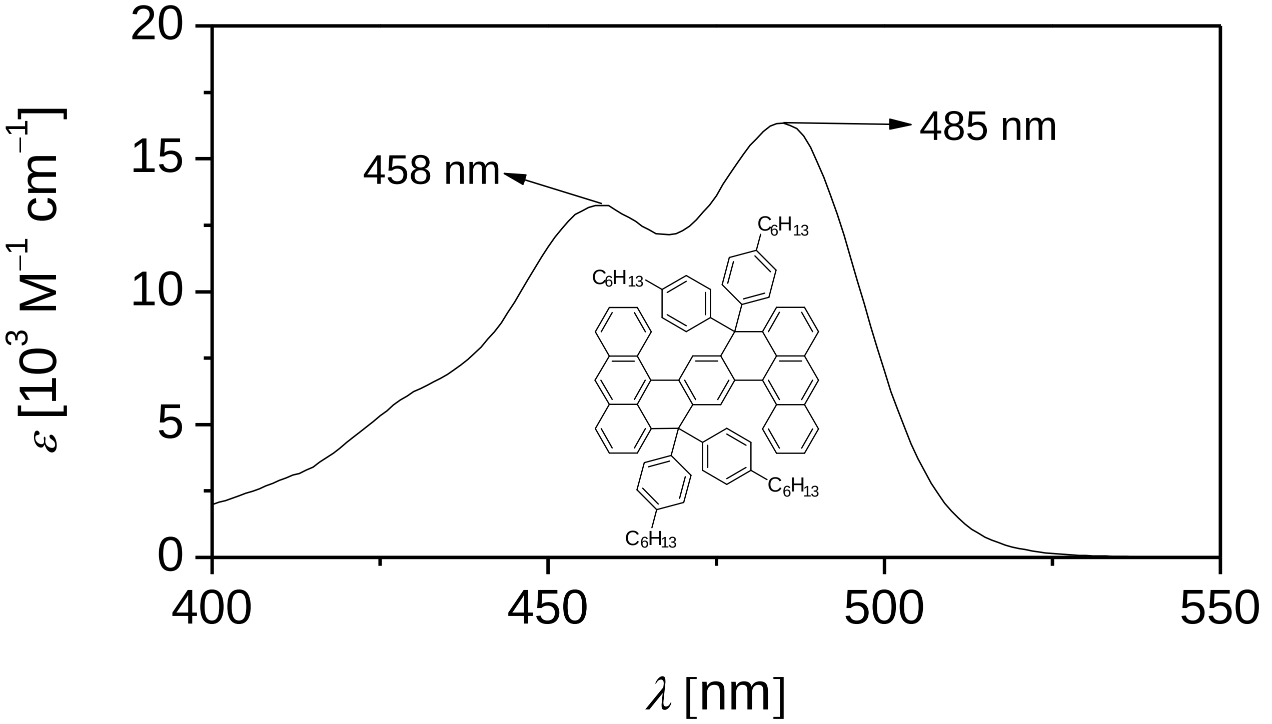

Figure S17. The UV-Vis spectroscopy of **4** in THF.

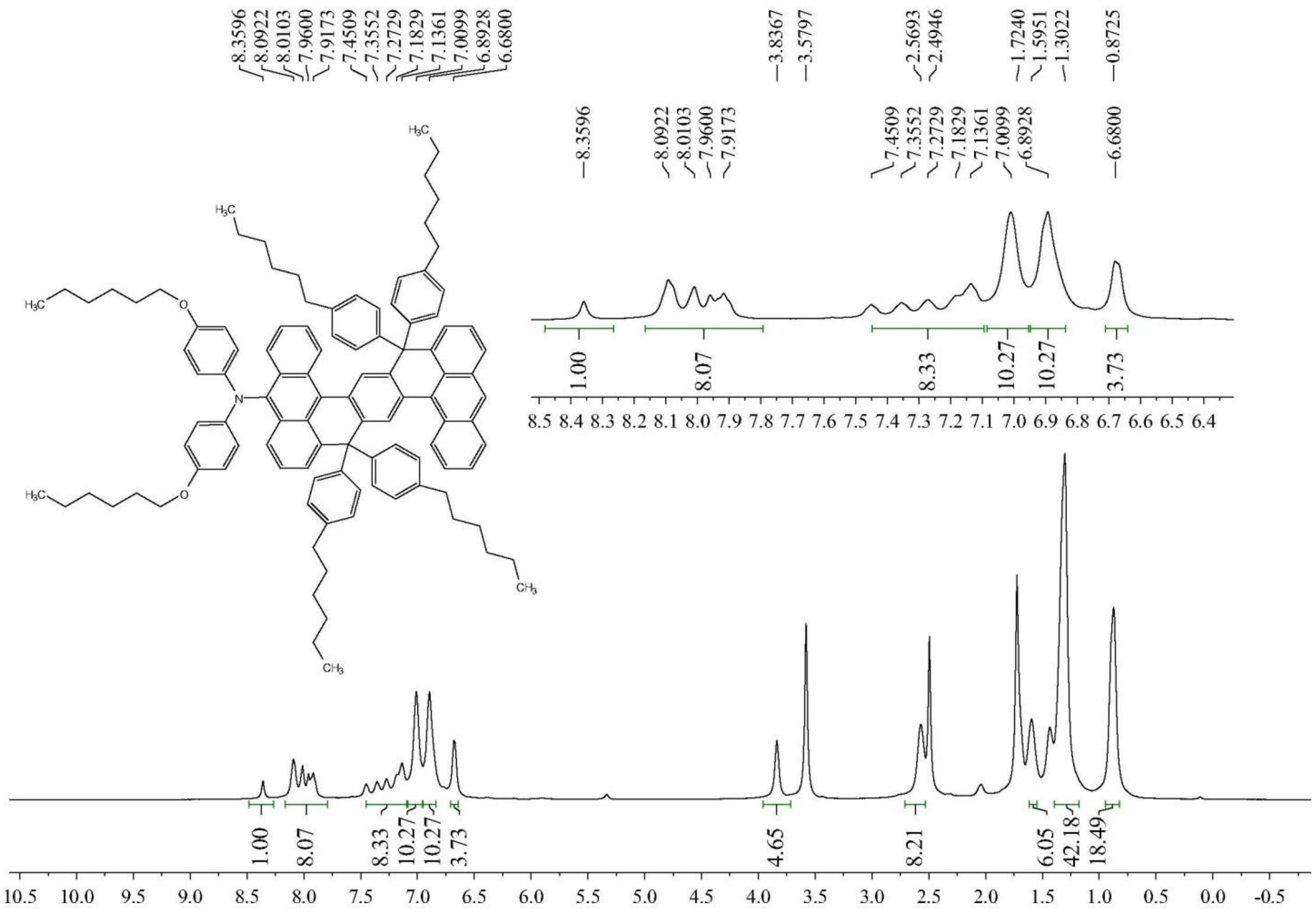

Figure S18. The ^{1}H NMR (500 MHz) spectrum of **7** in THF-d_8.

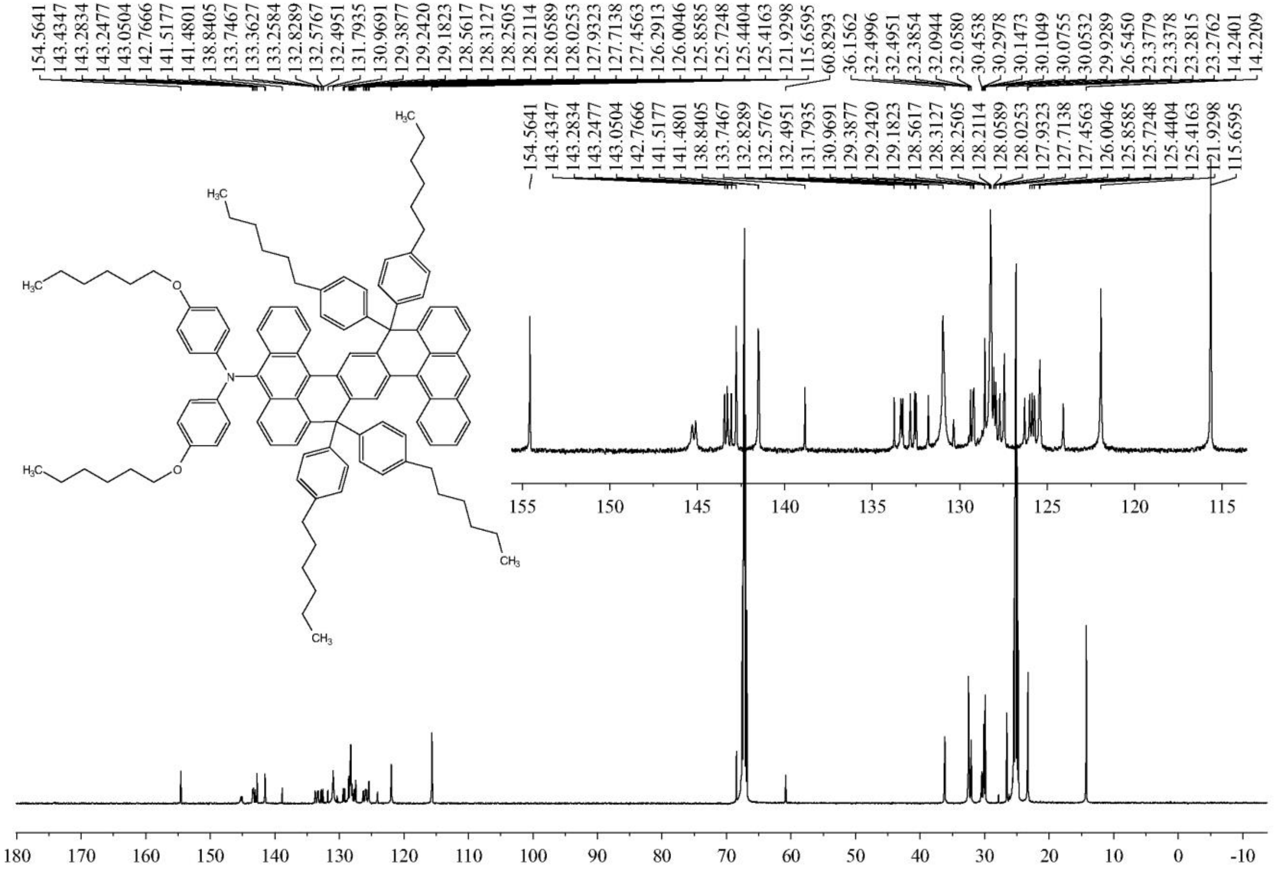

Figure S19. The ^{13}C NMR (125 MHz) spectrum of **7** in THF-d_8.

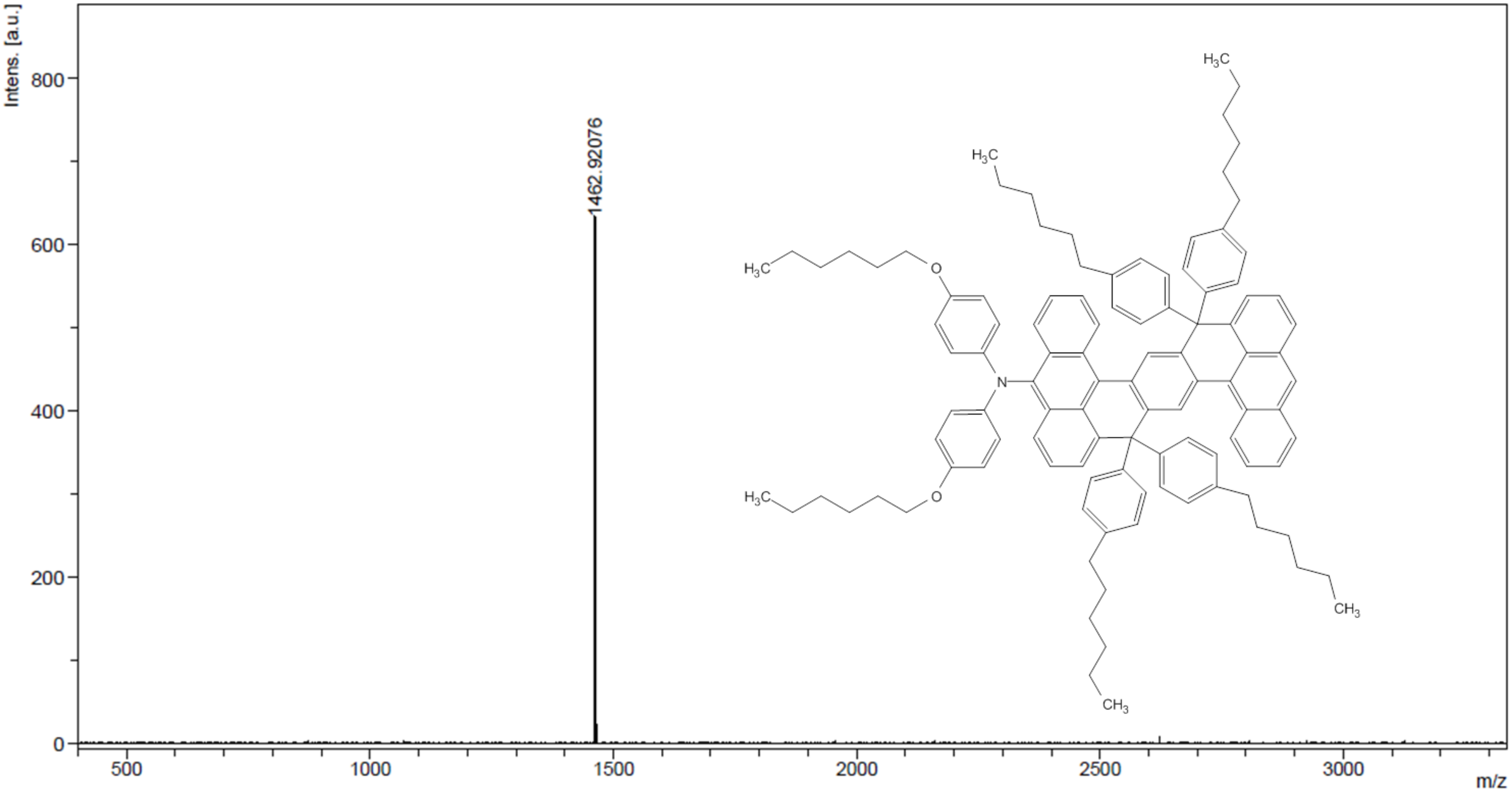

Figure S20. The high resolution mass spectrum (MALDI-TOF) of **7**.

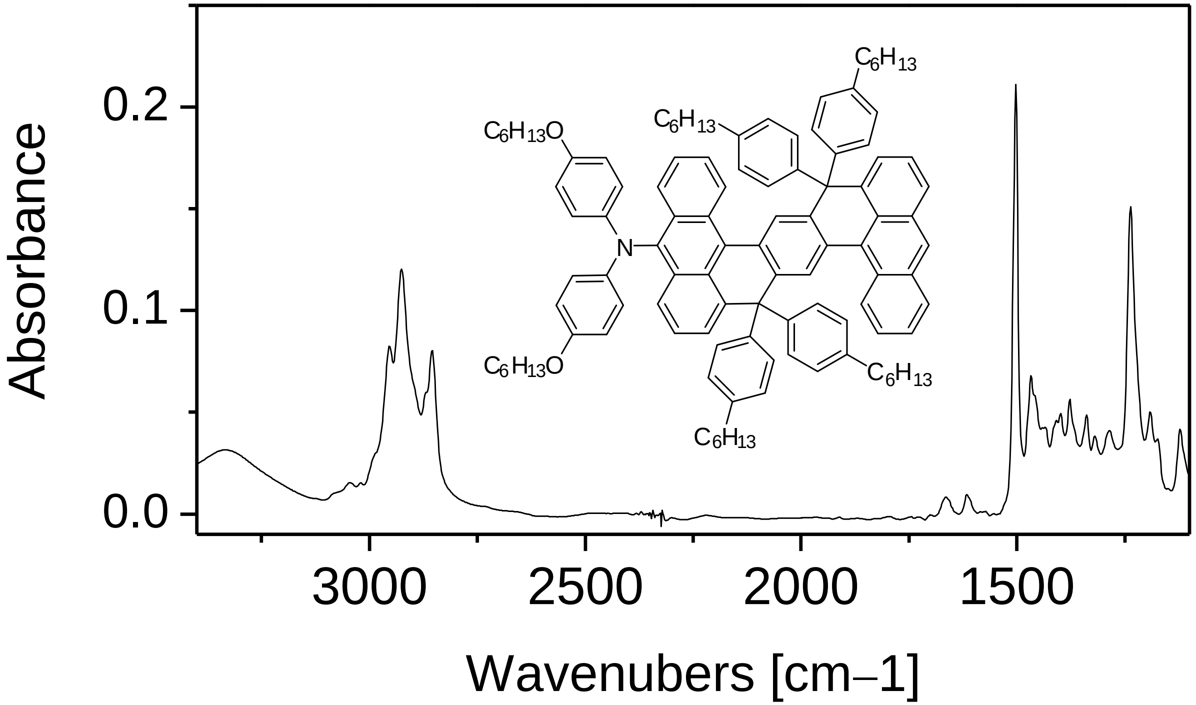

Figure S21. The ATR-FTIR spectrum of **7**.

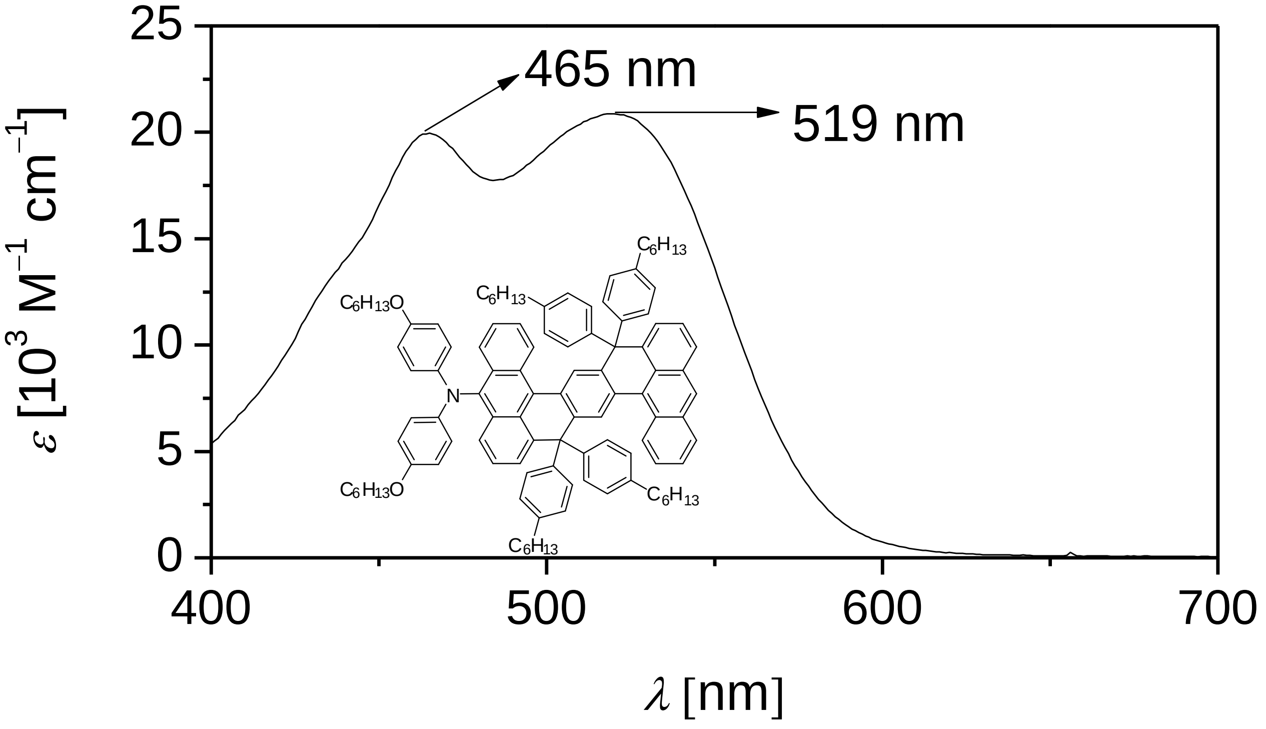

Figure S22. The UV-Vis spectroscopy of **7** in THF.

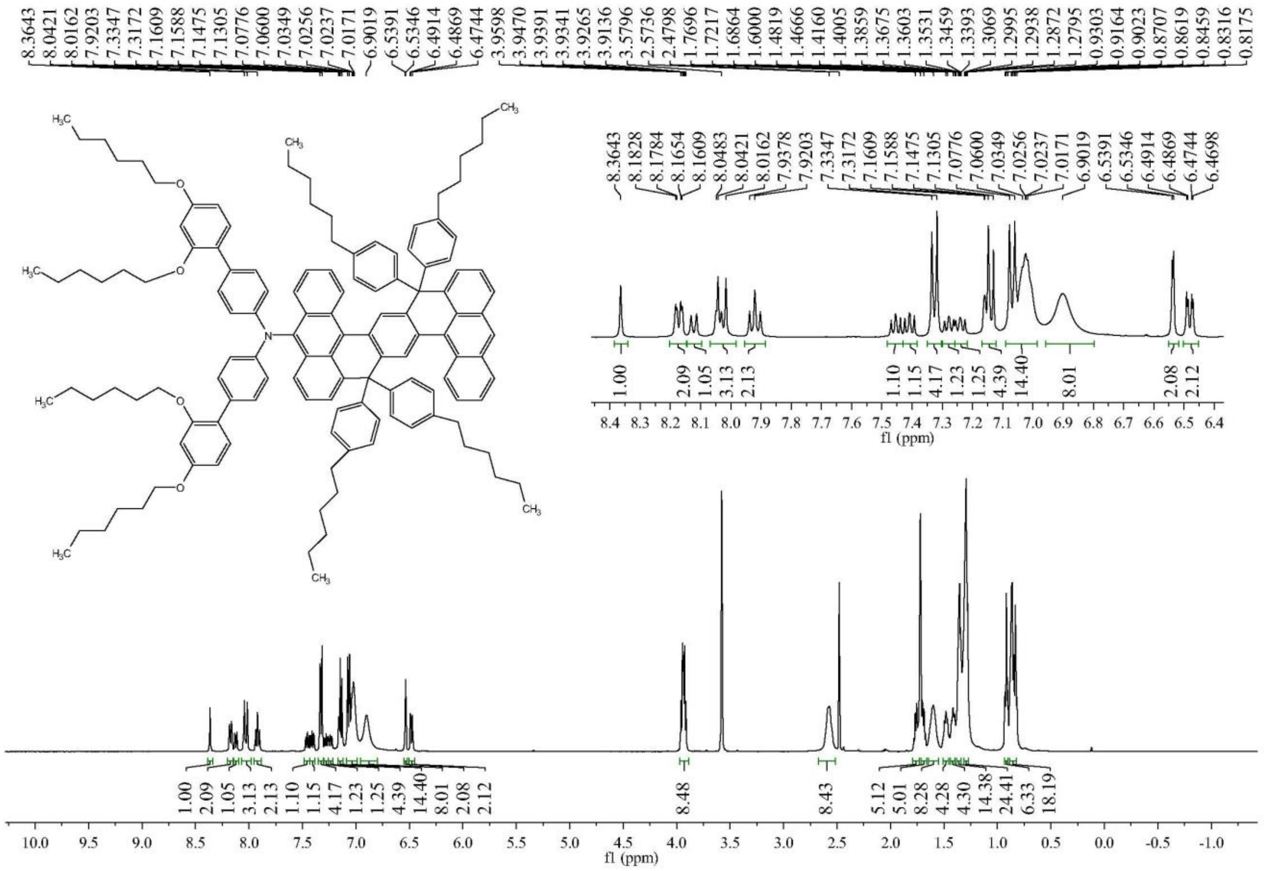

Figure S23. The ^{1}H NMR (400 MHz) spectrum of **8** in THF-d_8.

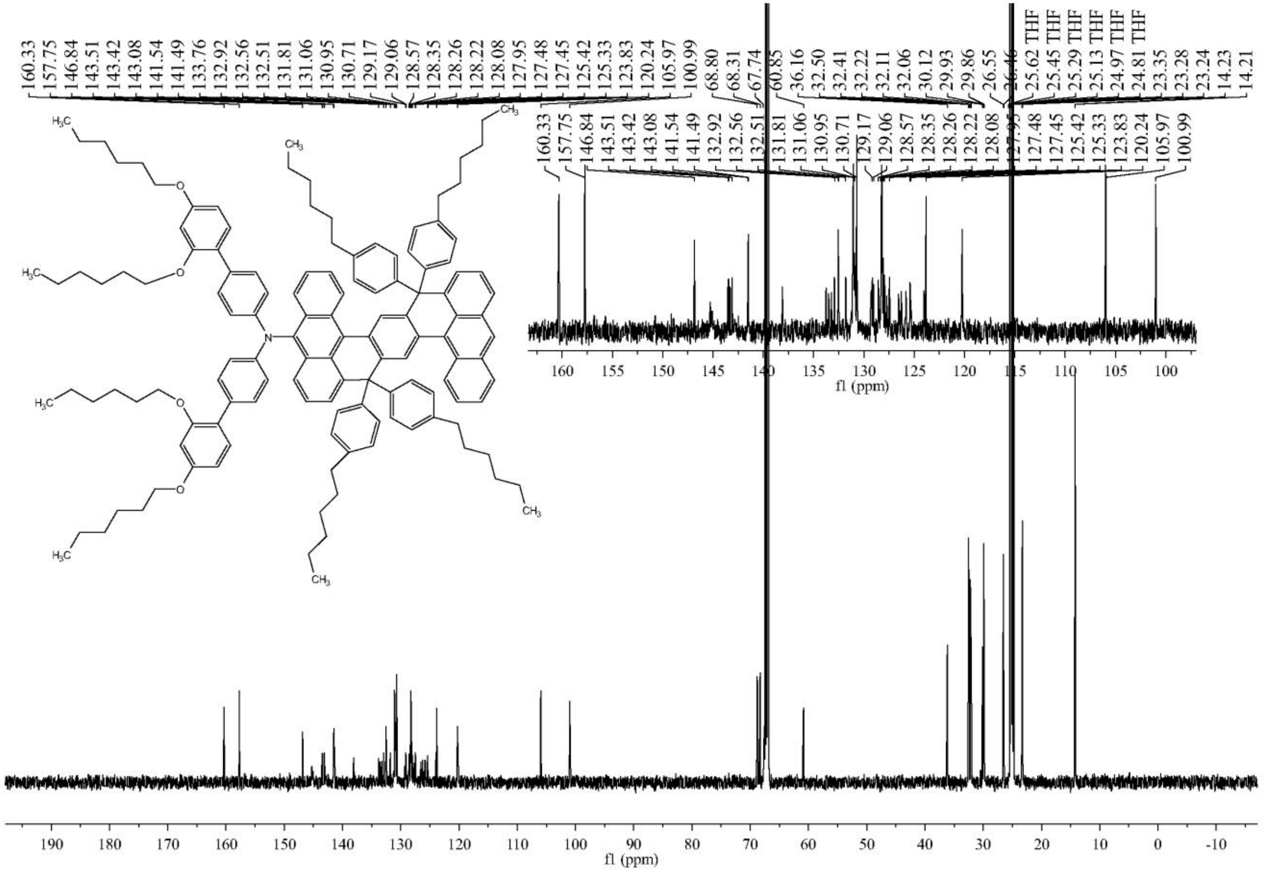

Figure S24. The ^{13}C NMR (100 MHz) spectrum of **8** in THF-d_8.

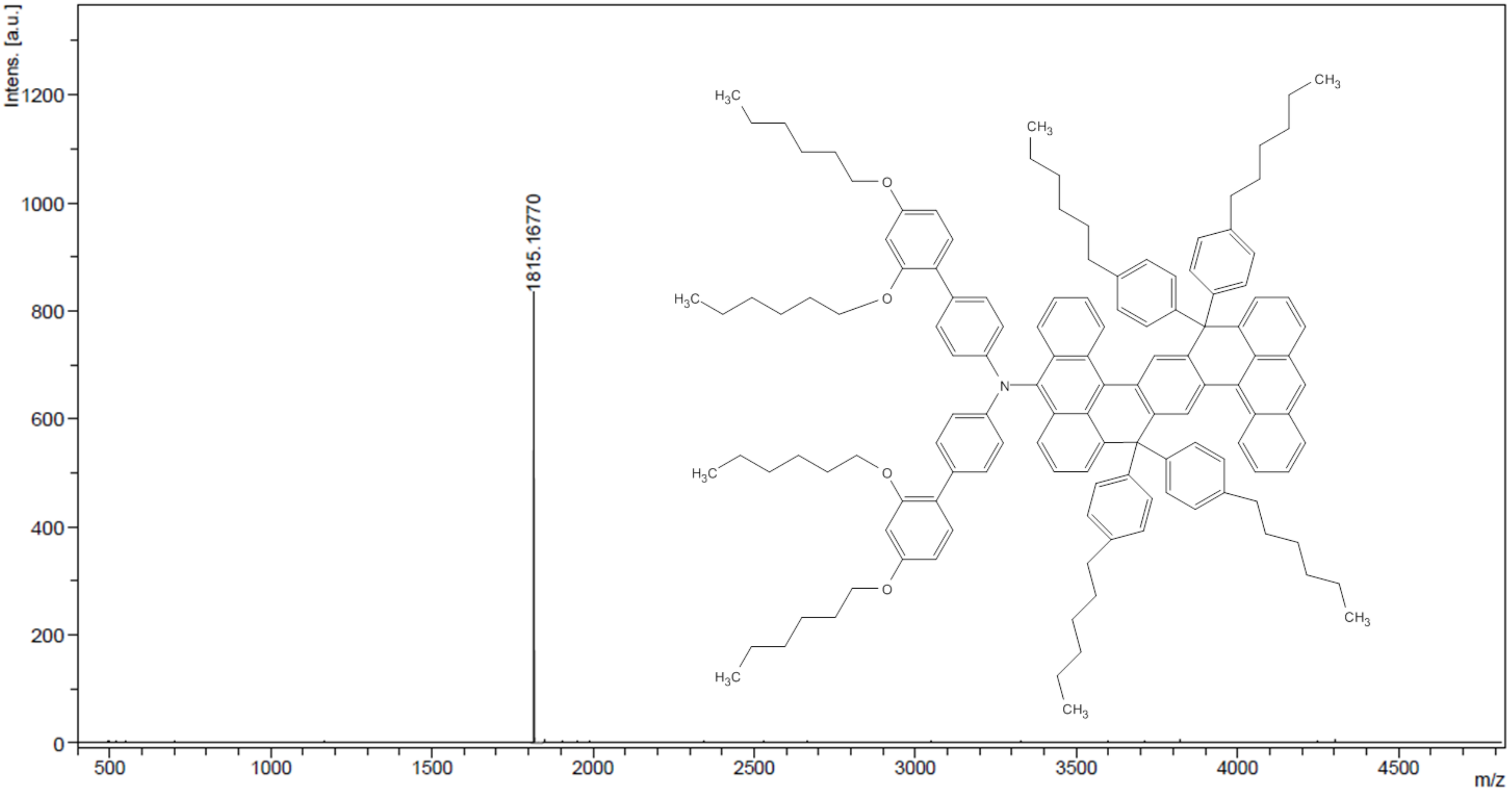

Figure S25. The high resolution mass spectrum (MALDI-TOF) of **8**.

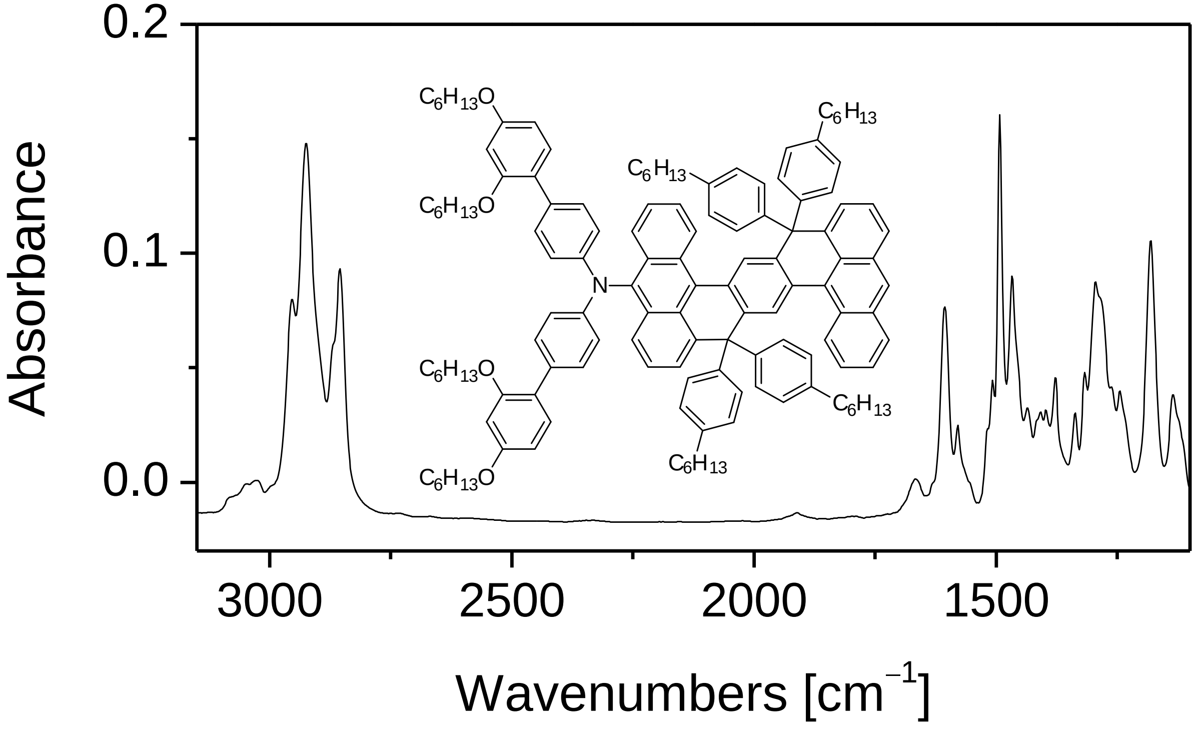

Figure S26. The ATR-FTIR spectrum of **8**.

.

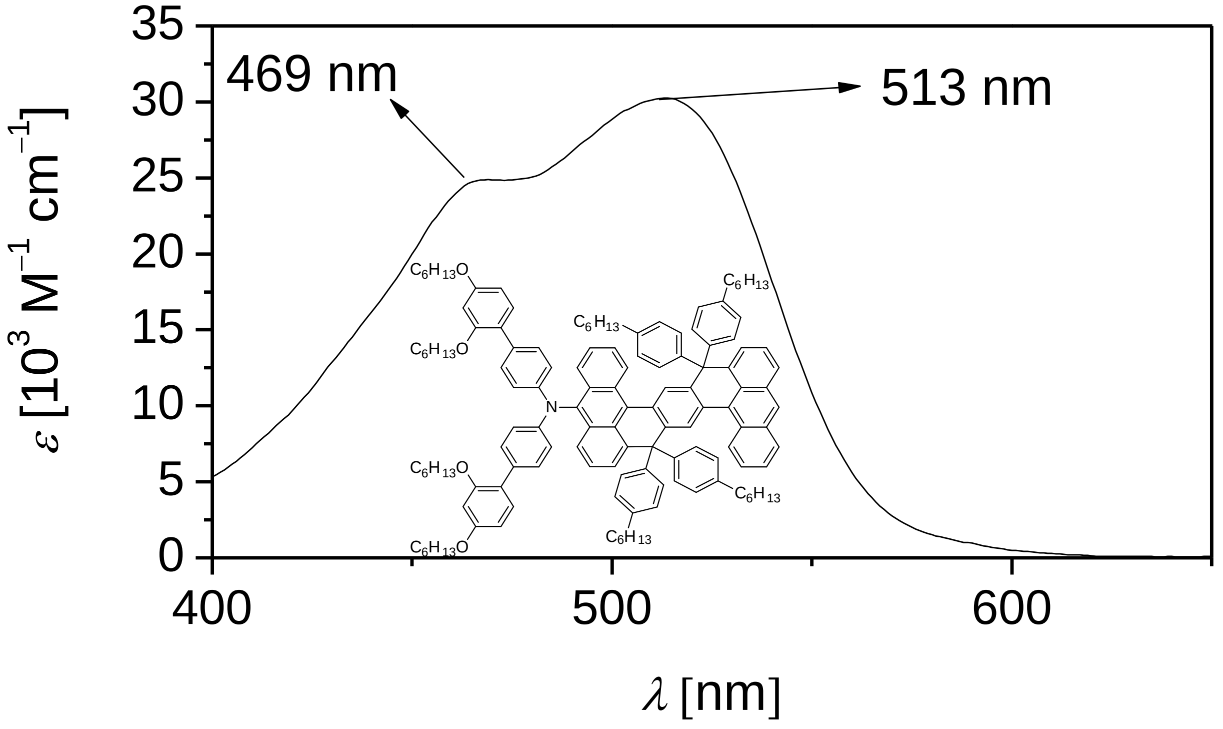

Figure S27. The UV-Vis spectroscopy of **8** in THF.

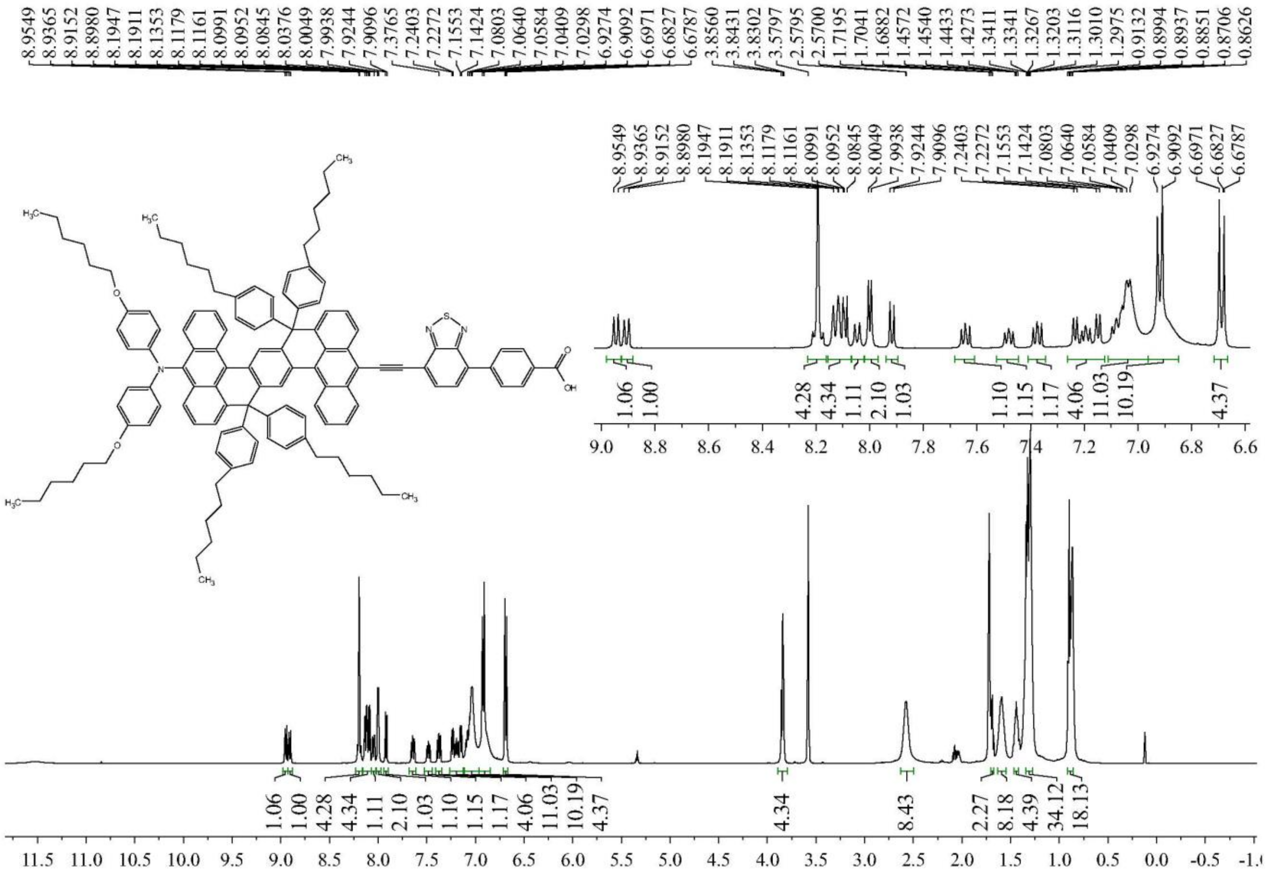

Figure S28. The ^{1}H NMR (500 MHz) spectrum of **R1** in THF-d_8.

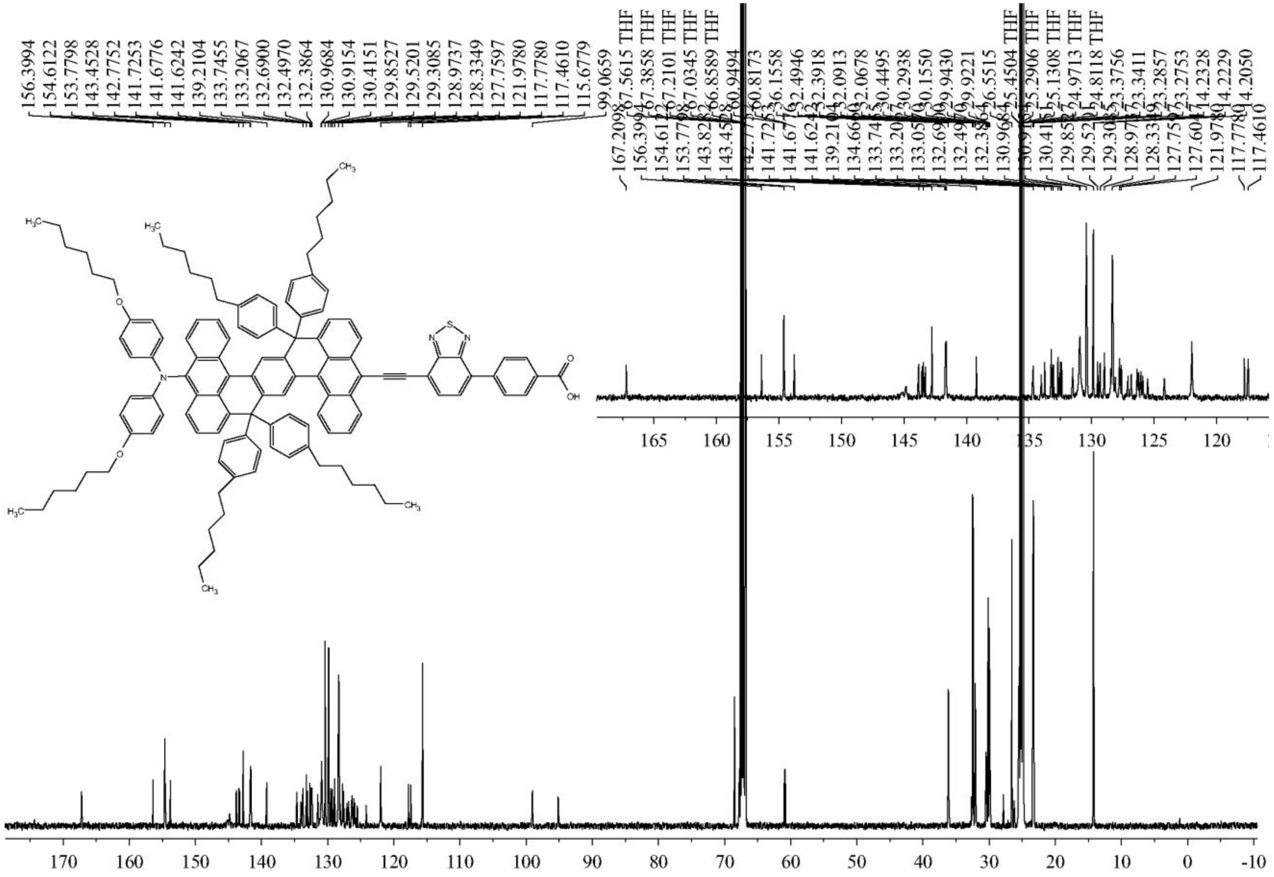

Figure S29. The ^{13}C NMR (125 MHz) spectrum of **R1** in THF-d_8.

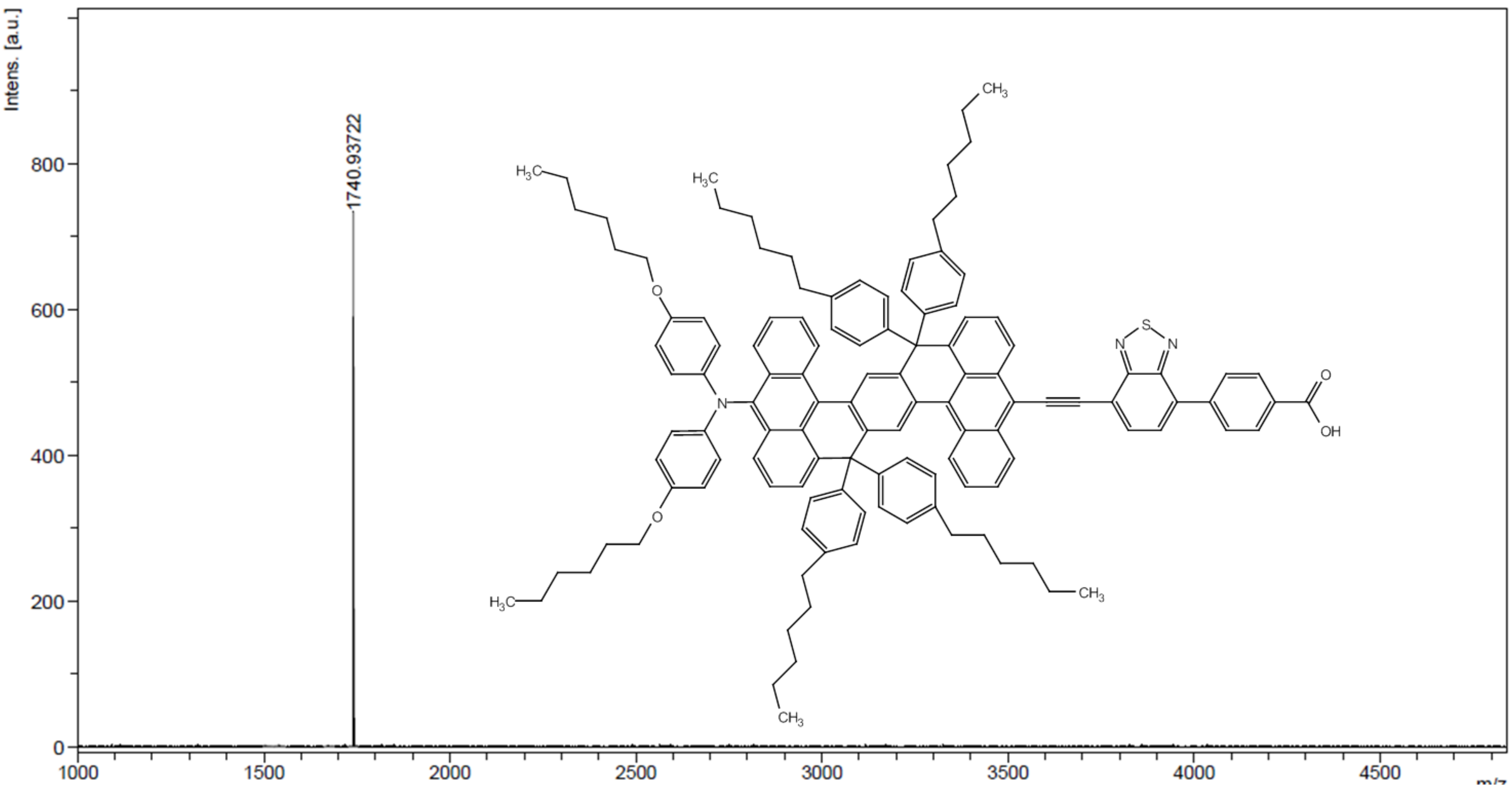

Figure S30. The high resolution mass spectrum (MALDI-TOF) of **R1**.

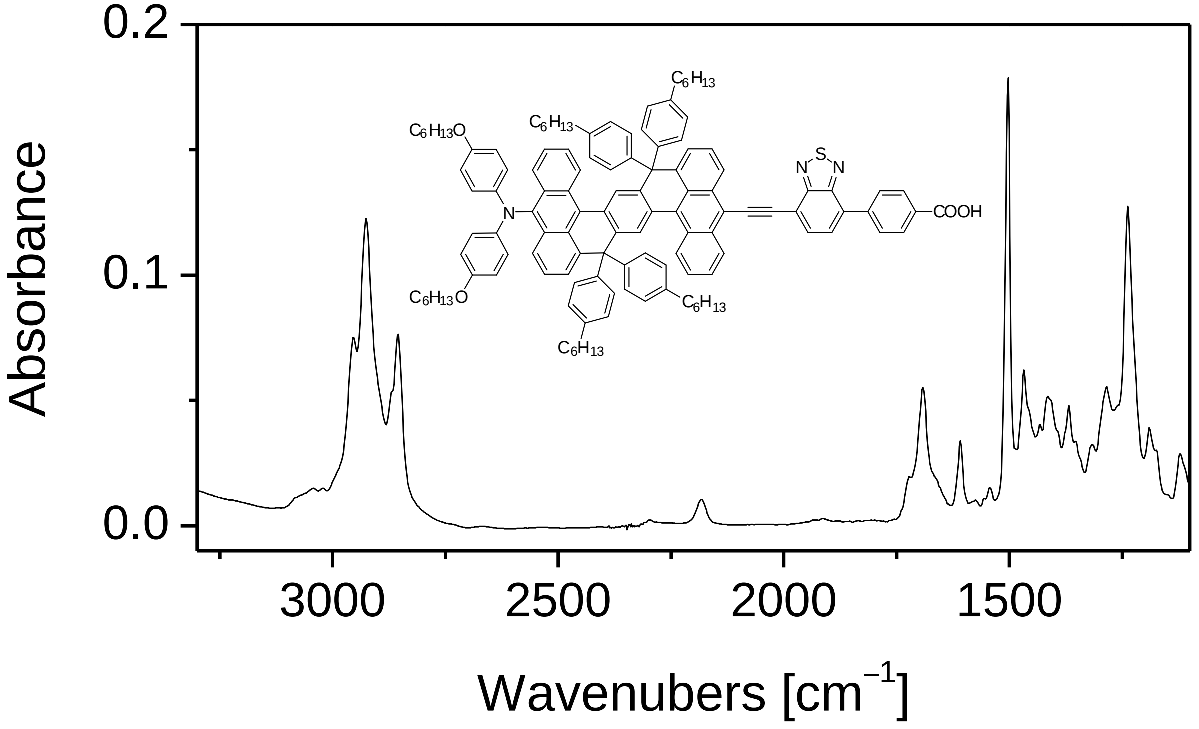

Figure S31. The ATR-FTIR spectrum of **R1**.

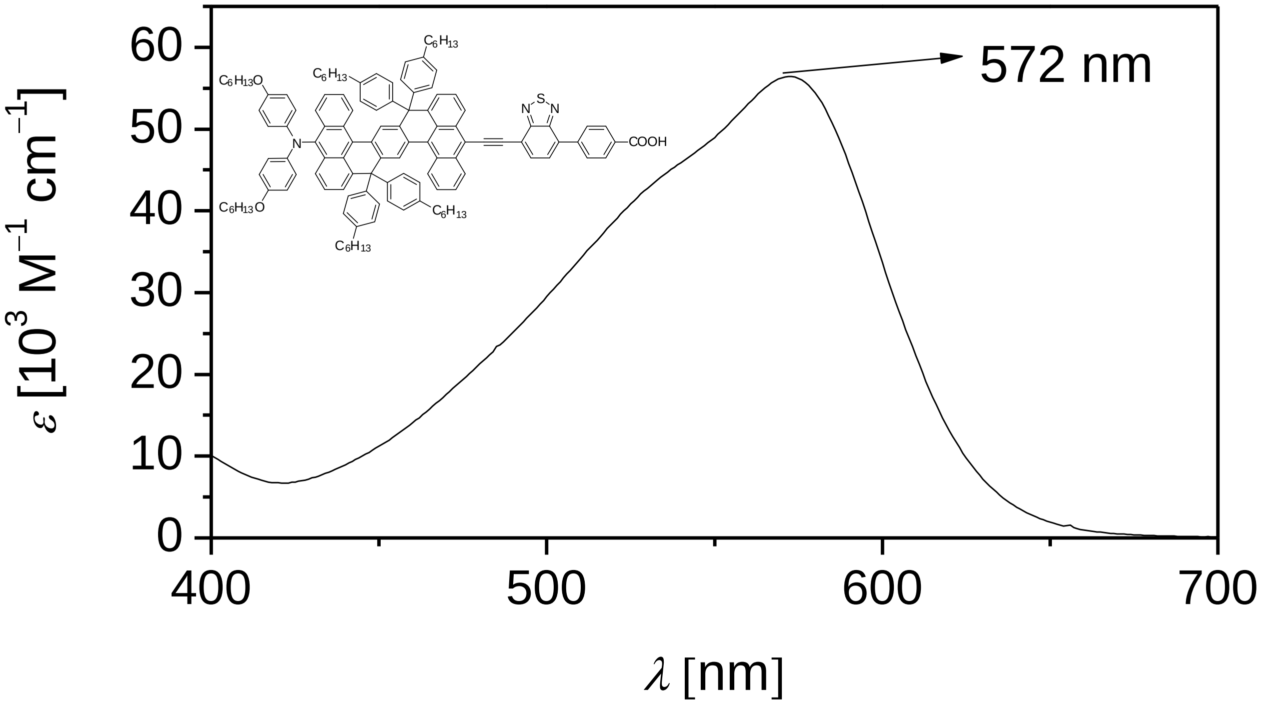

Figure S32. The UV-Vis spectroscopy of **R1** in THF.

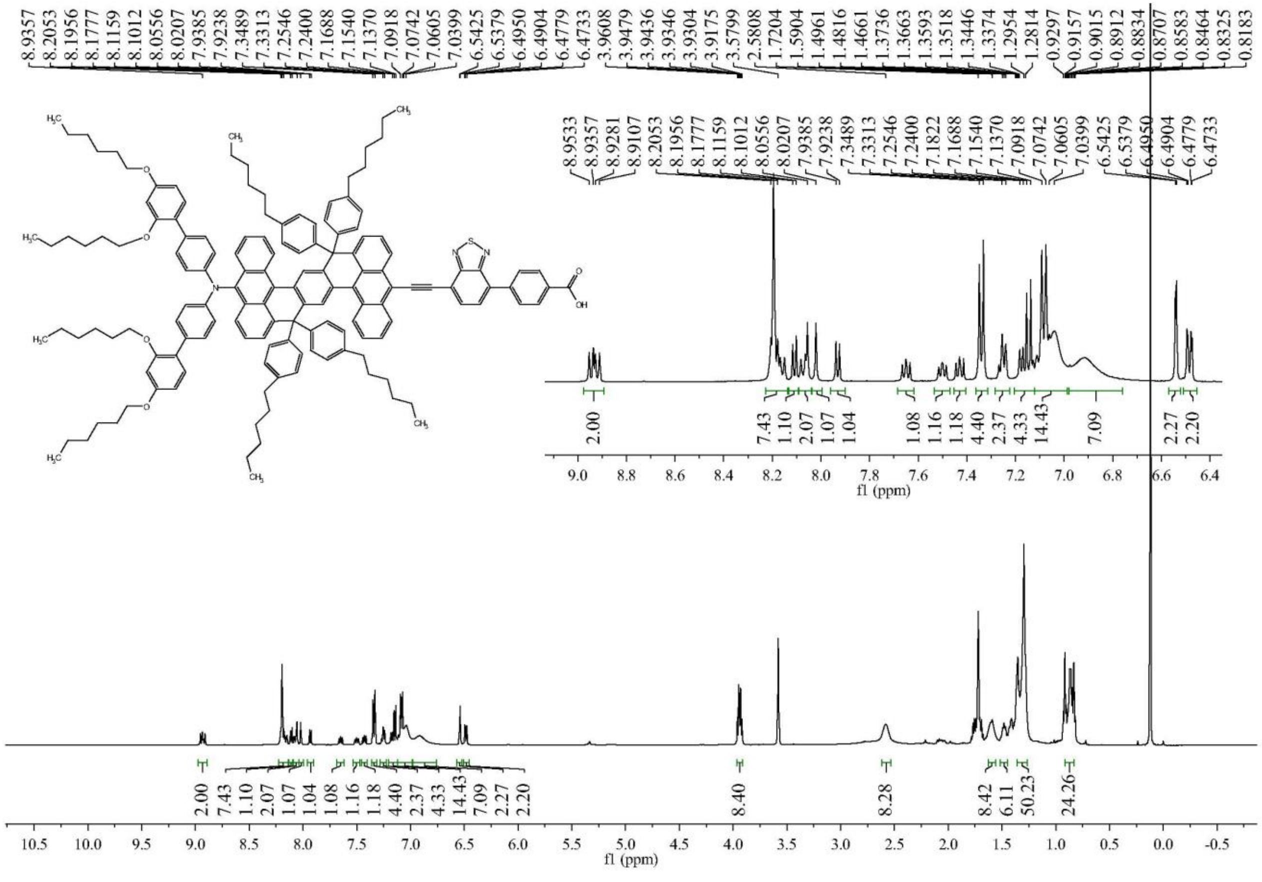

Figure S33. The ^{1}H NMR (500 MHz) spectrum of **R2** in THF-d_8.

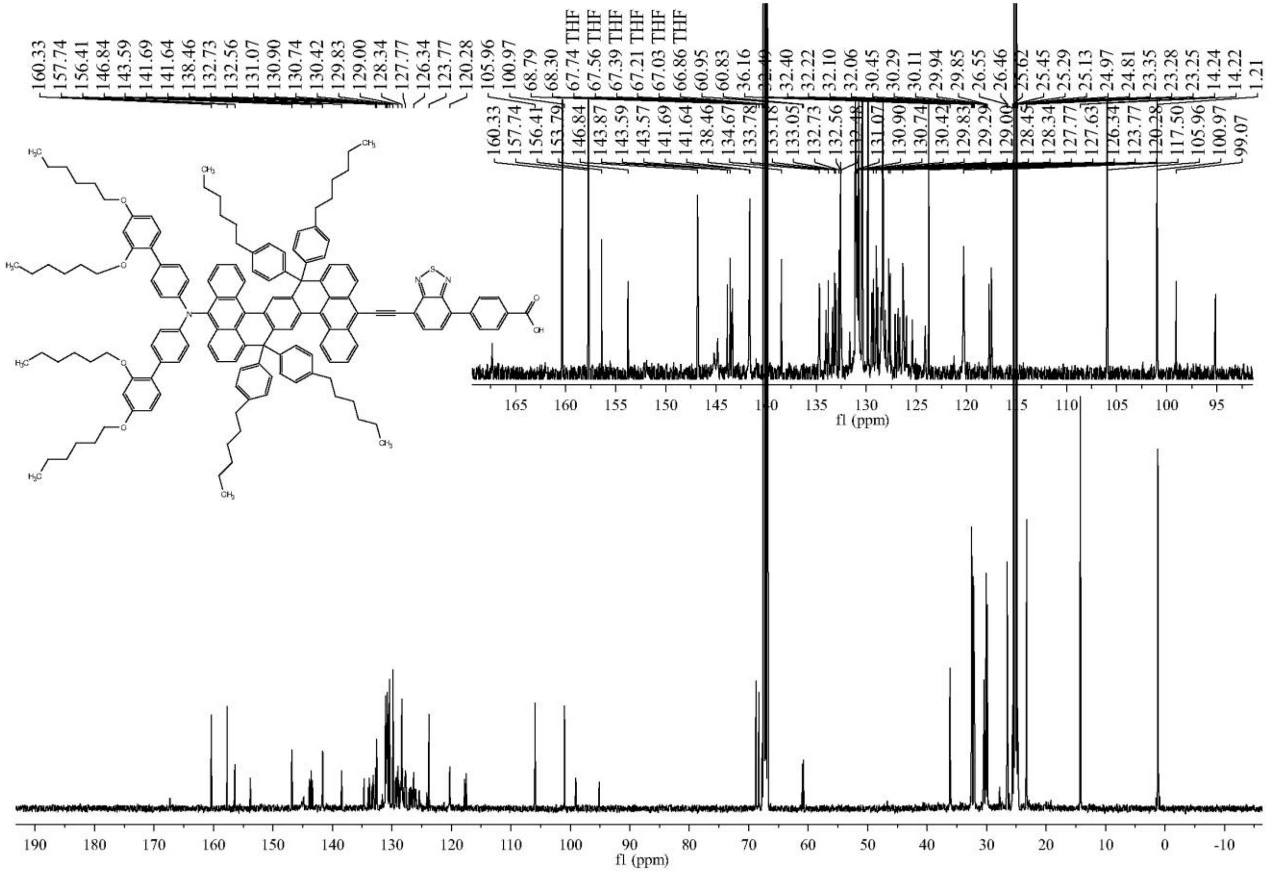

Figure S34. The ^{13}C NMR (125 MHz) spectrum of **R2** in THF-d_8.

.

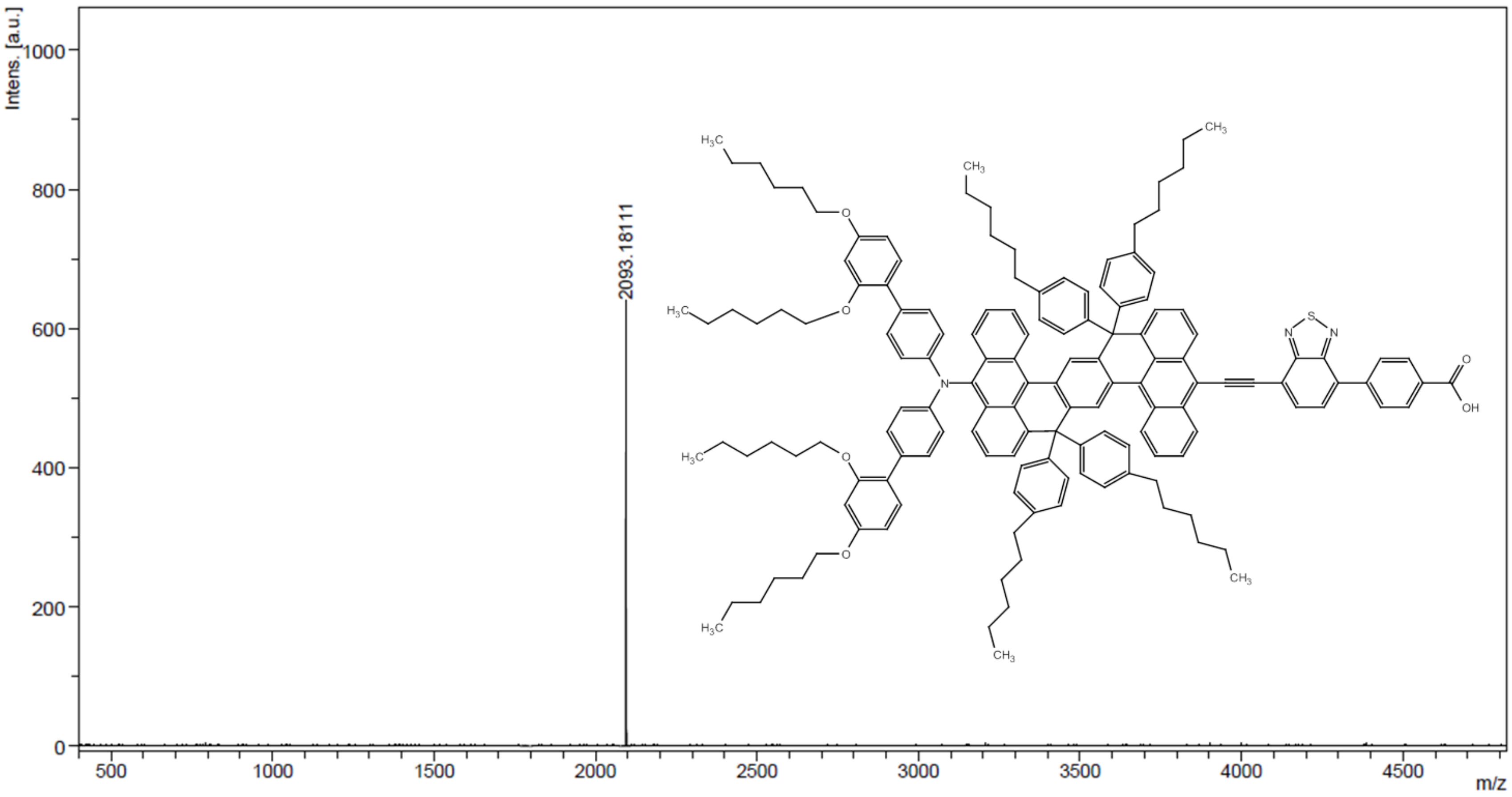

Figure S35. The high resolution mass spectrum (MALDI-TOF) of **R2**.

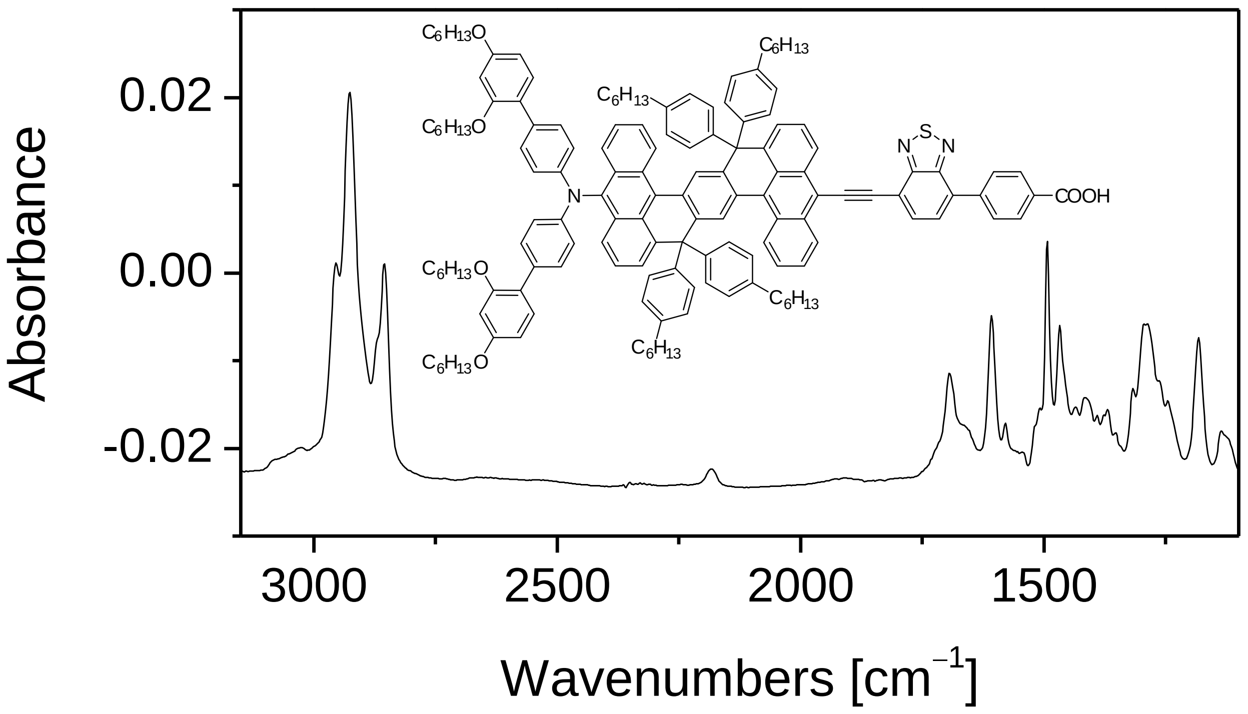

Figure S36. The ATR-FTIR spectrum of **R2**.

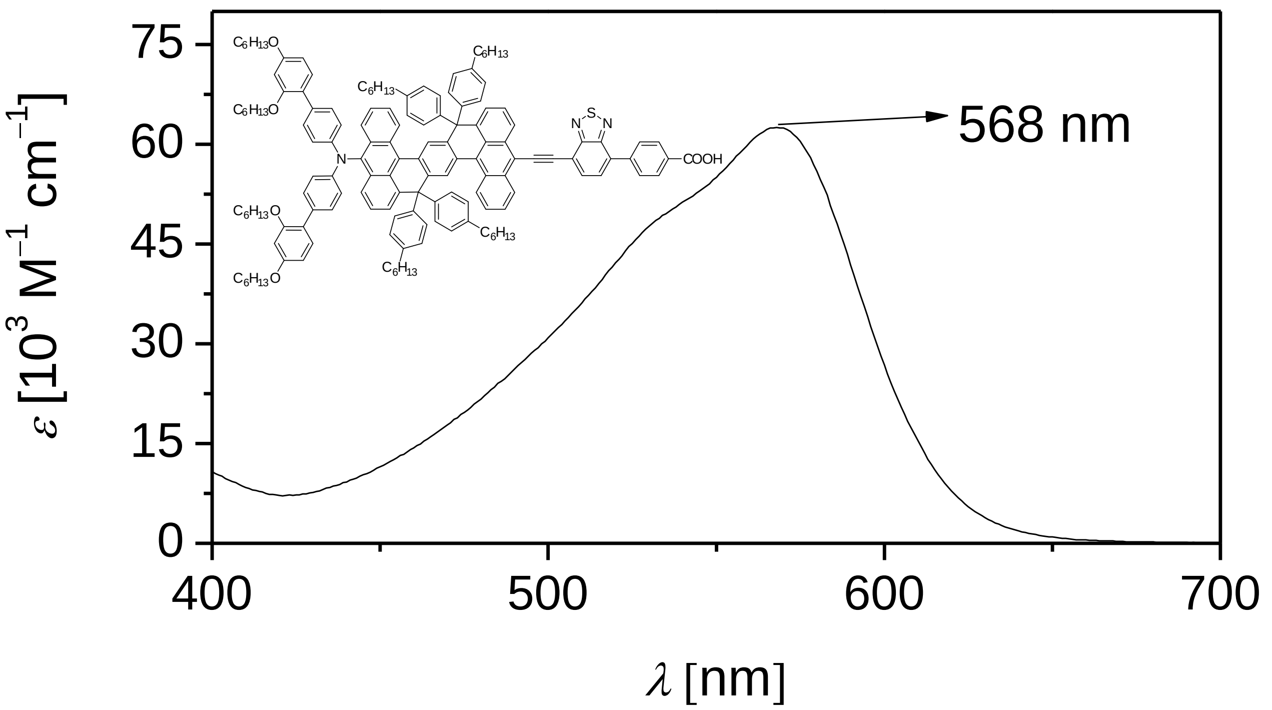

Figure S37. The UV-Vis spectroscopy of **R2** in THF.
